# Examination of the Peripheral Nervous System in Children With Spinal Muscular Atrophy: A High‐Resolution Ultrasonographic Study

**DOI:** 10.1002/brb3.71234

**Published:** 2026-02-05

**Authors:** Janina Wurster, Erin West, Sandro Meier, Noé Phillip Bürke, Lynn Jansen, Philip Julian Broser

**Affiliations:** ^1^ Children's Hospital of Eastern Switzerland St. Gallen Switzerland; ^2^ University of Zurich Zurich Switzerland; ^3^ Clinical Trials Unit HOCH Health Ostschweiz Kantonsspital St. Gallen St. Gallen Switzerland

**Keywords:** cross‐sectional area, HRUS, nerve structure, spinal muscular atrophy

## Abstract

**Objectives:**

Recent studies have shown that high‐resolution ultrasound (HRUS) devices allow us to accurately measure peripheral nerves in newborns. In consideration of these developments, this study aimed to analyze the structure and cross‐sectional area (CSA) of the median nerve in children with SMA and evaluate the usefulness and reproducibility of HRUS imaging for the monitoring of peripheral nerves in these children.

**Methods:**

A total of 12 participants aged 1–15 years with SMA were included in this repeated cross‐sectional study. In addition, 97 normally developing children aged 2 days to 17 years were included as controls. Using HRUS devices, the structure and CSA of the median nerve were determined at three sites (wrist, forearm, and above the elbow). The measured CSA and nerve structure were compared between the groups.

**Results:**

The CSA of the median nerve was smaller in the children with SMA than in the controls. Compared to the controls, SMA children had a mean CSA ranging from 0.70 to 1.02 mm^2^ smaller while adjusting for age. Similar to normally developing children, the increase in CSA with age in children with SMA can be described using a logarithmic curve. Furthermore, ultrasonographic examination indicated a loss of the fascicular structure of the nerves, which, together with muscle atrophy, led to an altered sonographic appearance and more difficult visualization.

**Conclusion:**

HRUS is a useful method for monitoring nerve growth in children with SMA.

## Graphical abstract

This study aimed to analyze the structure and cross‐sectional area (CSA) of the median nerve in children with spinal muscular atrophy (SMA) and evaluate the usefulness of high‐resolution ultrasound (HRUS) imaging for the monitoring of peripheral nerves in these children. A total of 12 children with SMA and 97 normally developing children were included in this repeated cross‐sectional study. The mean CSA of the median nerve, while adjusting for age, was smaller in the children with SMA (dots) than in the controls (red trend line). Furthermore, ultrasonographic examination indicated a loss of the fascicular structure of the nerves, which, together with muscle atrophy, led to an altered sonographic appearance. Repeated measurements demonstrated that HRUS is a useful method for monitoring nerve growth in children with SMA.

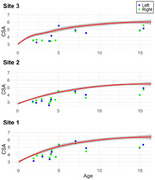

## 1 Introduction

Spinal muscular atrophy (SMA) is an autosomal recessive neuromuscular disease that leads to progressive muscle weakness and atrophy (Lunn and Wang 2008; Wadman et al. 2019). The incidence of SMA is approximately 1 per 6000–12,000 births (Wadman et al. 2019). If left untreated, it is one of the leading genetic diseases that cause death in childhood (Pearn 1980). Five subtypes of SMA (0–IV) are distinguished depending on the motor function achieved and the age of symptom onset; of these, Type I is the most common subtype (Lunn and Wang 2008; Russman 2007). Muscle weakness and atrophy are the result of the degeneration of anterior horn cells (Lunn and Wang 2008). In most SMA patients, this is due to a homozygous deletion of the survival motor neuron 1 (*SMN1*) gene (Lunn and Wang 2008). However, different studies have implied that structures other than motor neurons in the spinal cord are also affected by SMN protein deficiency (Lunn and Wang 2008; Wadman et al. 2019; Murray et al. 2008). Genetic analyses have shown that a second gene (*SMN2*) generates a small amount of functional SMN protein, even though it is slightly different from the protein produced by the *SMN1* gene (Calucho et al. 2018). Therefore, the SMN2 copy number is an important factor that influences the SMA phenotype (Calucho et al. 2018; Prior et al. 2000). This is also reflected in the analyses of Calucho et al. (2018), which clearly showed that a larger number of SMN2 copies is often associated with a milder phenotype.

Since the identification of the *SMN* gene as the disease‐causing gene in 1995, much progress has been made in the molecular understanding of SMA and the development of new treatment options (Lunn and Wang 2008). Three disease‐modifying therapies (DMTs) have been approved in recent years: nusinersen (Spinraza), onasemnogene abeparvovec (Zolgensma), and risdiplam (Evrysdi) (Erdos and Wild 2022).

To date, treatment success is determined using caregiver or self‐evaluations and various clinical scores such as the Children's Hospital of Philadelphia Infant Test of Neuromuscular Disorders or the Hammersmith Functional Motor Scale Expanded for SMA (Erdos and Wild 2022). The current gold standards for objective clinical information are electromyography and nerve conduction studies (Lunn and Wang 2008; Yonekawa et al. 2013). However, these examinations are associated with a significant burden for children and their parents, which contributes to their reluctance to be performed routinely.

Since recent studies have shown that HRUS devices allow us to accurately measure the peripheral nerves of children and newborns (Jenny et al. 2020), the aim of this study was to investigate the structure and cross‐sectional area of the median nerve in children with SMA and to evaluate the usefulness of high‐resolution ultrasound devices as objectifiable tools for the assessment and monitoring of peripheral nerves in these children.

## 2 Methods

From November 2023 to September 2024, this repeated cross‐sectional study was conducted at the Children's Hospital of Eastern Switzerland. The study was approved by the Ethics Committee of St. Gallen (EKOS approval no. 2019–02200), and all participants' caregivers provided written informed consent before the participants were included in the study.

A total of 12 children aged 1–15 years with SMA were included in this study. In addition, 97 normally developing children aged 2 days to 17 years were included as controls. The participants with SMA were assigned to the main study group, while the normally developing, term‐born children without severe acute or chronic diseases or neurological disorders were designated as the comparison (control) group. In the control group, the children, especially those between 0 days and 2 years of age, were examined with HRUS to precisely reflect nerve development during this period. This same control group was previously reported as the source of normative data in the study by Broser and Lütschg (2024).

To reduce the margin of error, a strict study protocol was followed for all examinations of the control and main study groups. Moreover, to minimize interrater variability, the median nerve was identified and traced solely by JW. JW's intrarater variability was tested on a sample of three children (aged 0, 4, and 16 years). For this purpose, the CSA was measured three times for each position and participant, and the maximum difference was calculated for Site 1 (0 years: 3.4%, 4 years: 2.8%, and 16 years: 3.6%), Site 2 (0 years: 3.9%, 4 years: 2.2%, and 16 years: 1.2%), and Site 3 (0 years: 3.1%, 4 years: 4.3%, and 16 years: 3.7%).

Ultrasound images were obtained using the Canon Aplio i800 system (Canon Medical Systems, Tokyo, Japan) with the i22LH8 ultrasound probe capable of a maximum scanning frequency of 22 MHz. If possible, additional scans were performed with the i33LX9 probe (33 MHz transducer) to visualize the nerve structure in more detail.

### 2.1 Ultrasound Imaging and Measurements

The median nerve was analyzed due to its easy accessibility and the established and validated HRUS measurement method, as previously described by Jenny et al. (2020).

**FIGURE 1:**
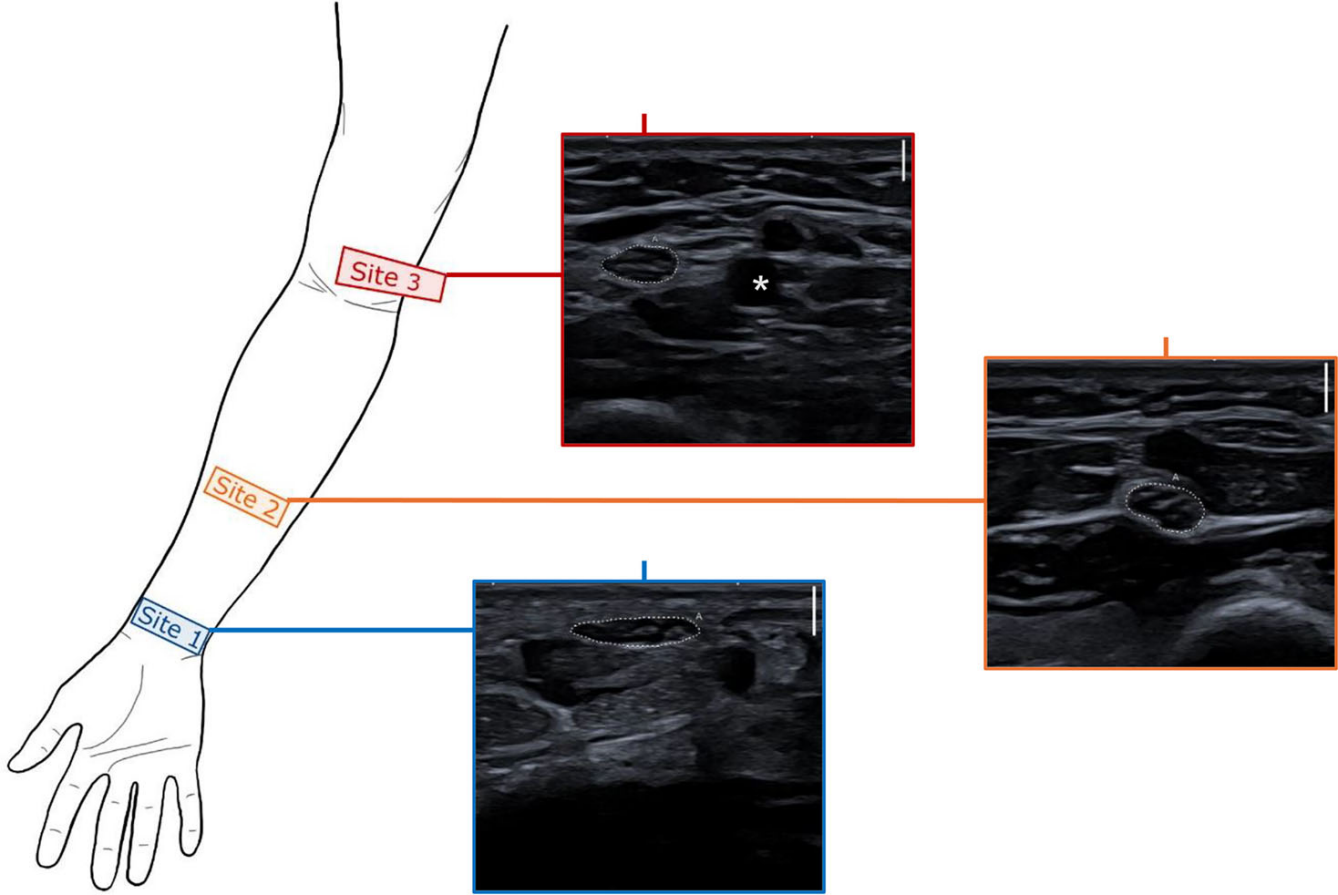
Schematic illustration of the three imaging sites. Site 1: At the level of the wrist, palmar, near the pronator quadratus muscle. Site 2: At the level of the forearm, midway between the elbow and the wrist, where the median nerve lies between the superficial and deep flexor muscles. Site 3: At the level of the upper arm, just proximal to the elbow, where the median nerve runs alongside the brachial artery (localized using ultrasound Doppler). Dotted line: the median nerve; (*): the brachial artery.

For the ultrasonographic examination, the arm was held in a supinated position. The median nerve was then visualized at three specific sites for each participant (Jenny et al. 2020; Peer and Gruber 2013): the wrist, forearm, and just above the elbow (Figure 1). Whenever possible, both arms were examined. However, in the comparison group, often only one arm could be scanned because of medical equipment (e.g., intravenous cannulas, casts).

To evaluate the reproducibility of HRUS measurements in children with SMA, repetitive measurements (2–4 US examinations per child) were performed in the main study group.

During the examination, short HRUS videos were recorded for each of the three sites, as the nerves of the participants with SMA were often difficult to identify. The course of the nerve was then traced on the recordings, and a still image was selected. The CSA (in square millimeters) and the circumference (in millimeters) were then measured in the stored still images using the freehand tracing tool of the Canon Aplio i800, as previously described by Jenny et al. (2020).

In addition to the measured values, further information of the participants, such as their sex, age, weight, height, and head circumference, was added to the study database. For the main study group, the current SMA therapy, the Hammersmith Functional Motor Scale (HFMS) score at the time of imaging and other factors that could influence nerve growth, such as SMN2 copy number, age of symptom onset, delay from symptom onset to the start of treatment, age at start of treatment, and therapy duration, were also documented in the study database (Tables 1 and 2a, 2b).

**TABLE 1:** Demographic data and growth parameters of the participants by disease status.

	Control	SMA
	(*N *= 97)	(*N *= 12)
Age (in years)		
Mean (SD)	6.15 (5.70)	6.21 (4.57)
Median [min, max]	4.53 [0.0100, 17.2]	4.37 [1.88, 15.4]
Sex		
Female	48 (49.5%)	8 (66.7%)
Male	49 (50.5%)	4 (33.3%)
Weight		
Mean (SD)	25.5 (21.4)	21.2 (8.72)
Median [min, max]	16.5 [2.63, 78.0]	18.5 [11.8, 37.1]
Height		
Mean (SD)	109 (41.5)	114 (25.2)
Median [min, max]	107 [46.5, 181]	104 [91.2, 161]
Head circumference		
Mean (SD)	44.4 (6.61)	51.5 (3.29)
Median [min, max]	44.0 [33.5, 57.5]	51.0 [44.4, 55.9]

*Note*: This table shows an overview of the demographics and growth parameters of the participants by disease status.

Abbreviations: N, number; SD, standard deviation.

**TABLE 2a:** Clinical and genetic characteristics of the children with SMA.

Therapy	
Evrysdi	1 (8.3%)
Spinraza	8 (66.7%)
Zolgensma	3 (25.0%)
Age at symptom onset (in years)	
Mean (SD)	0.873 (0.837)
Median [min, max]	0.833 [0.0416, 2.42]
Delay from symptom onset to treatment start (in years)	
Mean (SD)	1.88 (3.61)
Median [min, max]	0.178 [0.0136, 9.31]
Age at start of treatment (in years)	
Mean (SD)	2.41 (4.08)
Median [min, max]	0.285 [0.0384, 11.4]
Therapy duration (in years)	
Mean (SD)	3.80 (1.26)
Median [min, max]	3.82 [1.68, 5.85]
SMN2 copy number	
2 Copies	5 (41.7%)
3 Copies	5 (41.7%)
4 Copies	2 (16.7%)
SMA‐type based on age at symptom onset	
Type I	5 (41.7%)
Type II	3 (25.0%)
Type III	2 (16.7%)
Newborn screening	2 (16.7%)
Hammersmith Functional Motor Scale (HFMS) score	
SMA‐Type I (mean [min, max])	16.7 [12.0, 22.0]
SMA‐Type II (mean [min, max])	34.7 [13.0, 52.0]
SMA‐Type III (mean [min, max])	58.0 [54.0, 62.0]
2 SMN2 copies (mean [min, max])	16.7 [12.0, 22.0]
3 SMN2 copies (mean [min, max])	44.6 [13.0, 62.0]
4 SMN2 copies (mean [min, max])	53.0 [52.0, 54.0]

*Note*: For the 12 participants with SMA, this table provides information on the current therapy, age of symptom onset, start and duration of treatment, SMN2 copy number, and Hammersmith Functional Motor Scale (HFMS) score.

Abbreviations: N, number; SD, standard deviation.

**TABLE 2b:** Detailed clinical and genetic characteristics of the children with SMA.

ID	Age (years)	Sex	Age at symptom onset (months)	SMA type	Genetics	SMN2 copies	HFMS score	Current therapy
1	15.4	F	29	III	Homozygous deletion SMN1 gene	3	62	Spinraza
2	7.1	F	10	II	Homozygous deletion SMN1 gene	4	52	Spinraza
3	2.9	M	Screening		Homozygous deletion SMN1 gene	3	59	Spinraza
4	4.1	F	0.5	I	Homozygous deletion SMN1 gene	2	22	Zolgensma
5	14.9	F	14	II	Homozygous deletion SMN1 gene	3	13	Evrysdi
6	8.4	F	18	III	Homozygous deletion SMN1 gene	4	54	Spinraza
7	2.3	M	Screening		Homozygous deletion SMN1 gene	3	50	Zolgensma
8	4.6	F	2	I	Homozygous deletion SMN1 gene	2	12	Spinraza
9	1.9	F	1	I	Homozygous deletion SMN1 gene	2		Zolgensma
10	5.0	M	17	II	Homozygous deletion SMN1 gene	3	39	Spinraza
11	3.9	F	1	I	Homozygous deletion SMN1 gene	2	16	Spinraza
12	4.0	M	3	I	Homozygous deletion SMN1 gene	2		Spinraza

*Note*: This table provides a detailed insight into the SMA population and allows a comparison of demographic data, genetics, and clinical data.

Abbreviations: F, female; HFMS, Hammersmith Functional Motor Scale; M, male; SMN2, survival of the motor neuron 2 gene.

### 2.2 Statistical Analysis

**FIGURE 2:**
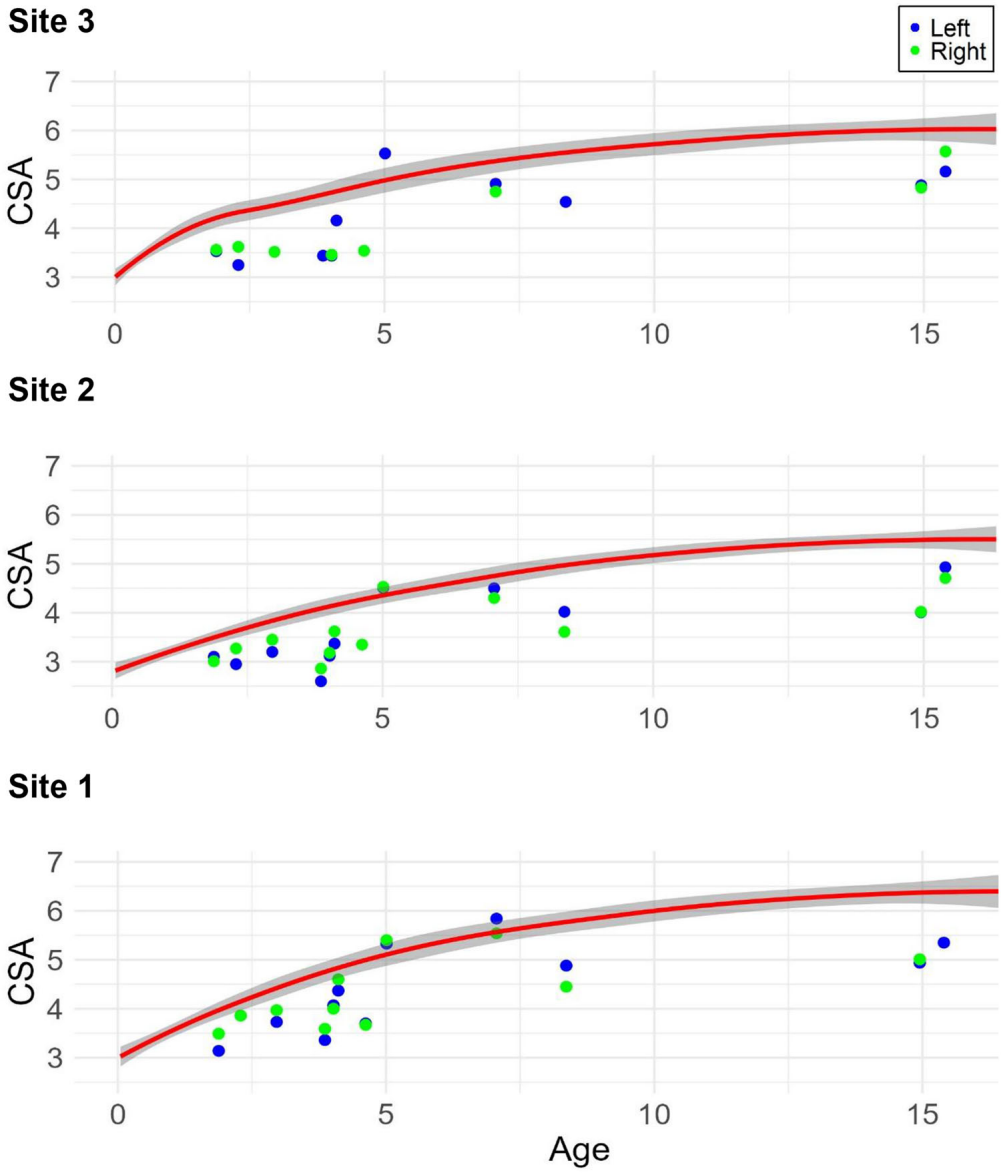
Scatterplots of the CSAs and ages for the control and SMA groups. Scatterplots of the CSAs in mm^2^ of the median nerve at Sites 1, 2, and 3 (left and right arms) and ages in years. The control group is represented by the trend line in red with the confidence interval in gray. The measurements of the SMA group are represented by blue dots (left arm) and green dots (right arm).

To analyze the growth behaviors of the median nerves in the SMA and control groups, scatterplots that compared age and CSA were generated. Given that it was often only possible to examine one arm in the control group owing to medical equipment, the CSA was plotted for both the left and right arms in the same graph to obtain representative values for all age groups (Figure 2).

To further investigate differences in nerve CSA between children with SMA and controls, we performed a linear regression model of the CSA at each location, with age and disease group as the independent predictors to determine the difference between the groups. Considering that the CSA more than doubles during childhood, we adjusted for age, as is often done in pediatrics (Jenny et al. 2020; Broser and Lütschg 2024). We report the mean differences and their 95% confidence intervals with the control group as the reference to show whether the SMA population has a smaller CSA while adjusting for the log of age (Table 3).

**TABLE 3:** Linear regression of the CSAs of the median nerves.

	Left arm		Right arm	
	Mean difference (95% CI)	*p* value	Mean difference (95% CI)	*p* value
Site 1	− 0.96 ( − 1.35 to − 0.56)	< 0.001	− 0.82 ( − 1.22 to − 0.41)	< 0.001
Site 2	− 1.02 ( − 1.35 to − 0.69)	< 0.001	− 0.74 ( − 1.04 to − 0.45)	< 0.001
Site 3	− 0.90 ( − 1.27 to − 0.54)	< 0.001	− 0.70 ( − 1.14 to − 0.25)	0.003

*Note*: Shown is the linear regression of the CSAs of the median nerves in mm^2^ at sites 1–3 for both arms, with log(age) and disease status as the independent predictors. An age adjustment was made considering that the CSA more than doubles during childhood. The table contains the mean differences and their 95% CIs for the disease variable for comparison between the SMA and control group, with the control group as reference.

The *p* value was obtained using Wald tests.

Abbreviation: CI, confidence interval.

In addition, other factors that could influence nerve growth, such as SMN2 copy number, age of onset of the first symptoms, and delay in treatment initiation, were evaluated graphically. However, in view of the small number of cases, statistical subgroup analyses were not considered useful.

Furthermore, a brief post‐hoc analysis was carried out on the relationship between head circumference (as an indicator of the development of the central nervous system) and age. This analysis was performed to rule out a general delay in the maturation of the nervous system. The corresponding scatterplot can be found in the Appendix A of Supporting Information. All analyses were performed using R version 4.3.2 and the ggplot2 package (R Core Team 2023; Wickham 2016).

### 2.3 Multiple Comparisons

The significance level *α* for the study was set to 0.05. To adjust for multiple statistical tests in this study, we performed a Bonferroni adjustment by dividing the *p* value of 0.05 by the number of statistical tests performed. Given that six statistical tests were performed in this study on the linear regression models of CSA across the two study groups, we set the corrected significance level *α* to 0.008.

## 3 Results

**FIGURE 3:**
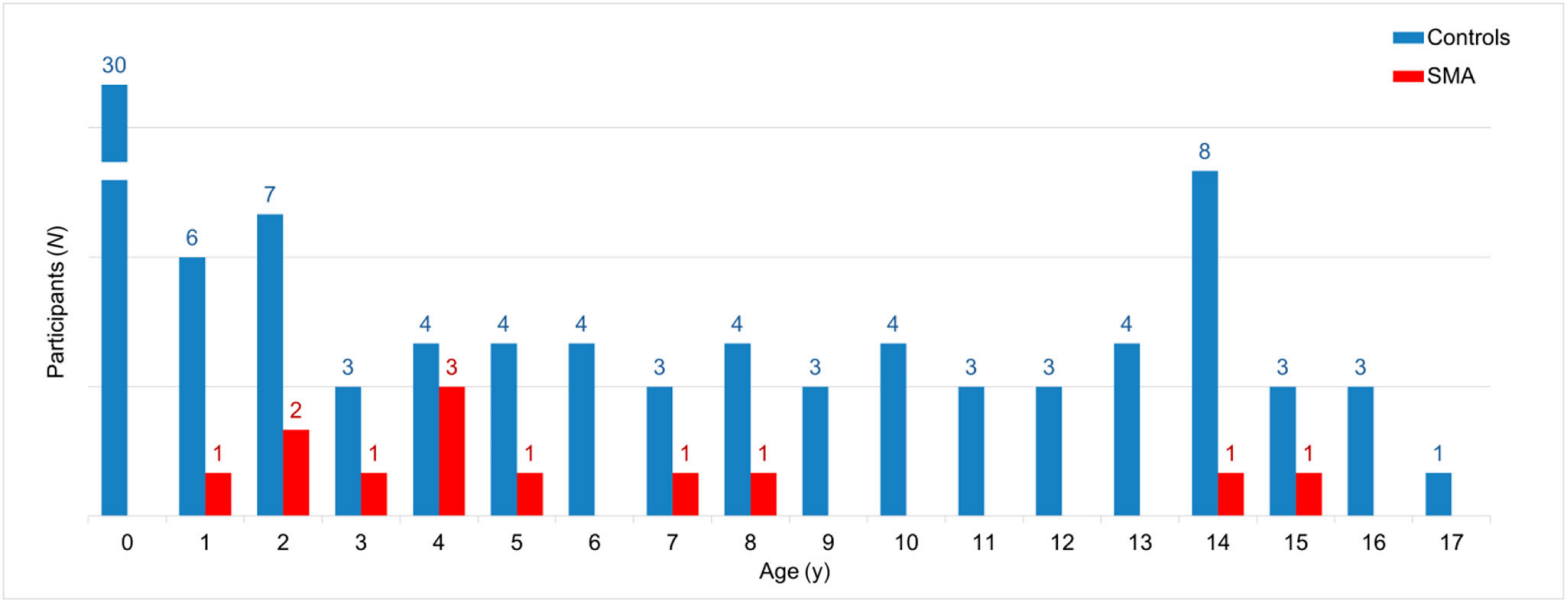
Age distribution of the study population. For better readability, the *y*‐axis (number of participants) was broken.

Twelve children with SMA were examined in this study. In addition, 100 normally developing children were examined as controls, of which three had to be excluded from the study because of underlying neurological conditions or incorrectly stored images. Finally, the study included 97 controls (49 males) aged between 2 days and 17 years. The age distributions of the children in the main study and control groups are shown in Figure 3. Additional demographic information and medical data are listed in Tables 1 and 2a, 2b.

### 3.1 Ultrasound Imaging

The subjective analysis of the dynamic ultrasonographic examination indicated a loss of the fascicular structure of the nerves in children with SMA compared to healthy controls, which, together with muscle atrophy, led to an altered sonographic appearance and more difficult visualization and delineation of the nerves. Figure 4a,b shows the age‐matched ultrasound images of a patient with SMA compared with those of a participant in the control group. Corresponding still images were used to measure the CSA of the median nerve in all study participants. The measurement results are reported in Appendix C of Supporting Information. Importantly, only ultrasound images with clearly distinguishable nerves were selected for CSA measurements. Therefore, structural changes are less impressive in these images.

FIGURE 4Exemplary ultrasound images. Each scale bar at the top right of the ultrasound images corresponds to a scanning depth of 2 mm. L stands for the left arm, and numbers 1–3 indicate the examination sites. (a) Cross sections of the median nerve of a patient with SMA at age 14.9 years and 3 months later. Corresponding images of a child from the control group at age 15.7 years are also shown. (b) Cross sections of the median nerves of a patient with SMA at age 4.6 years and 6 months later. The corresponding images of a child from the control group at age 4.5 years are also shown. (c) The series of images (Cross sections site L2 (forearm)) shows the loss of fascicular structure and poor demarcation of the nerve in a patient with SMA.

**Figure:**
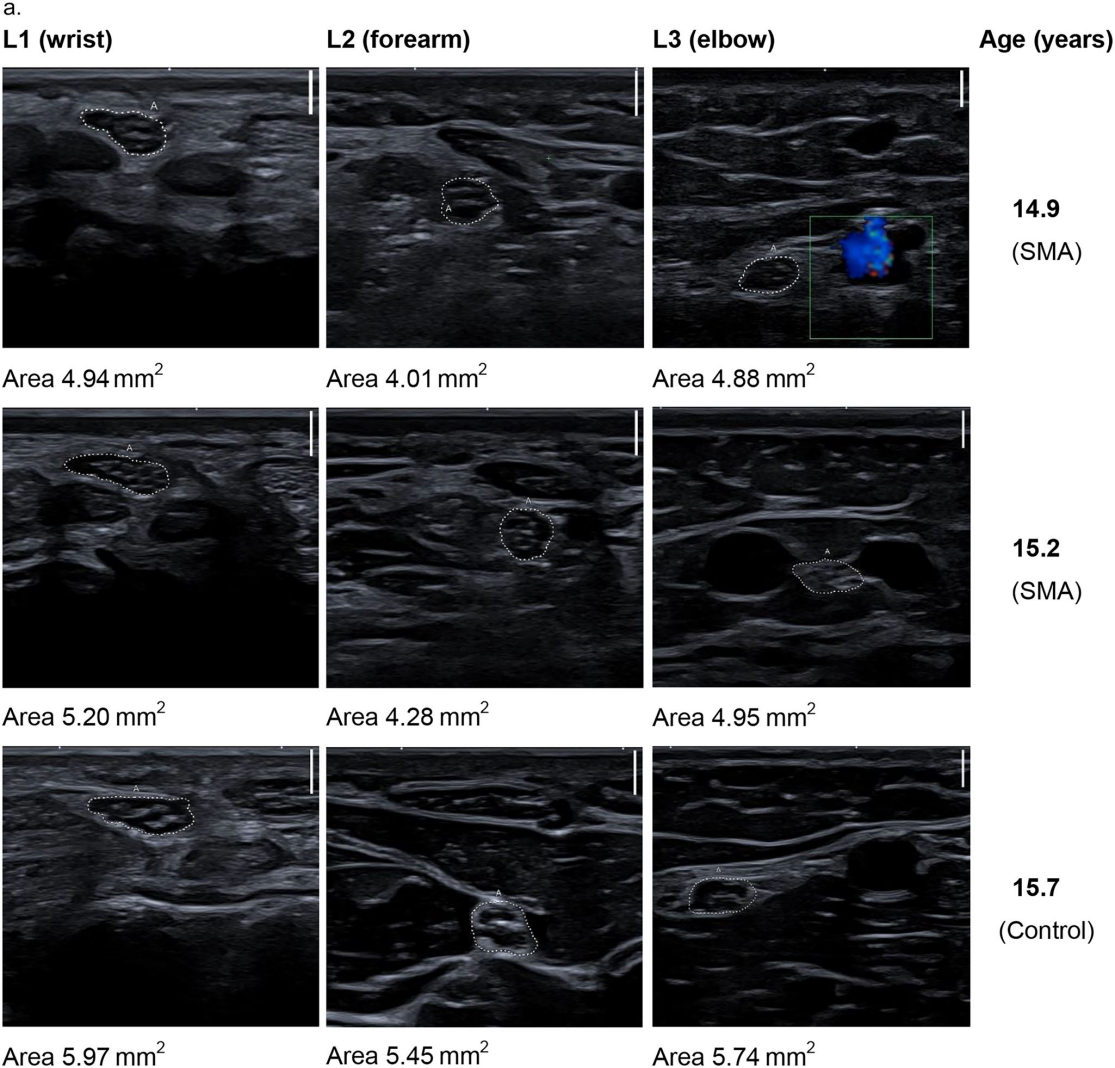

**Figure:**
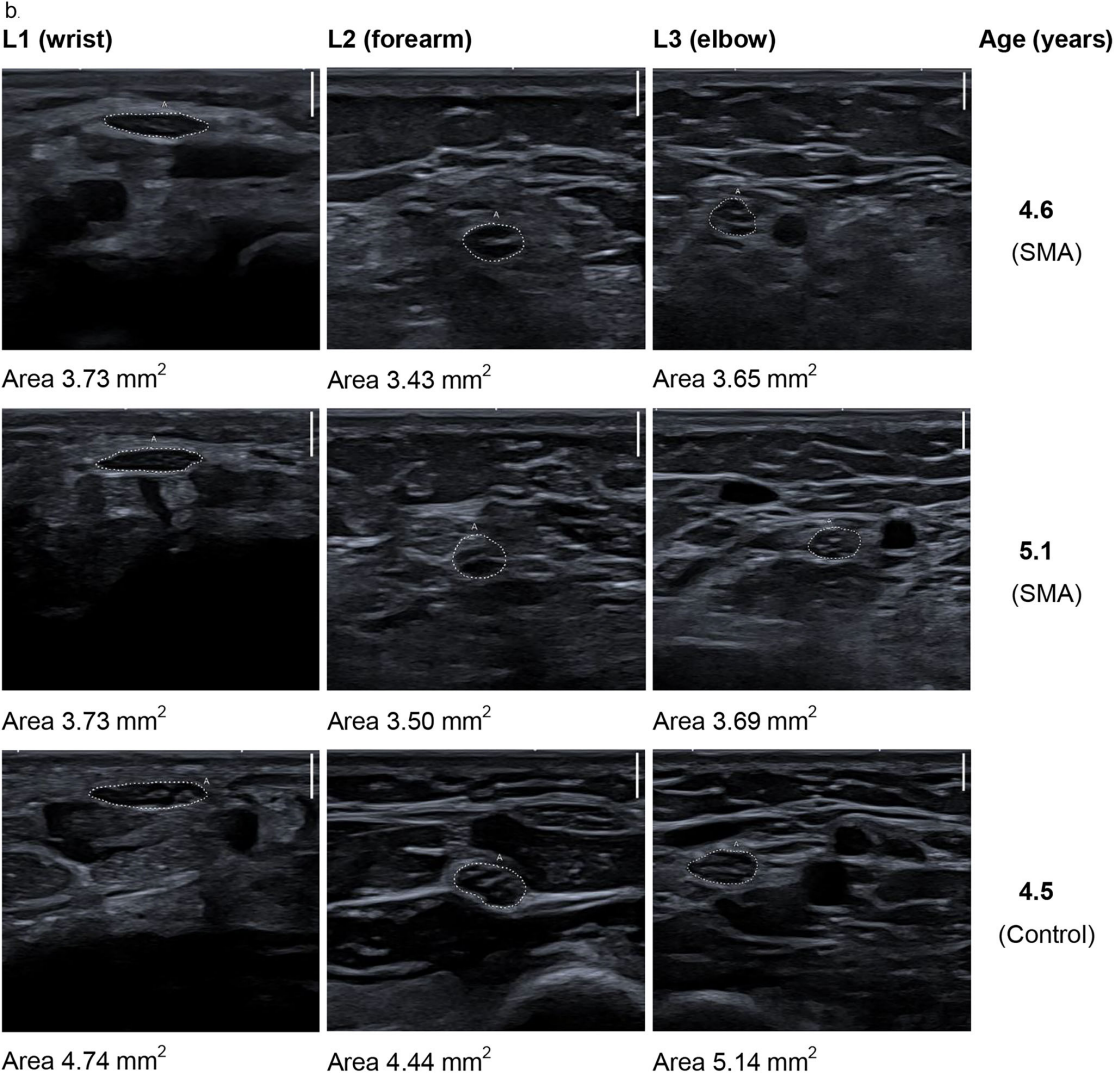

**Figure:**
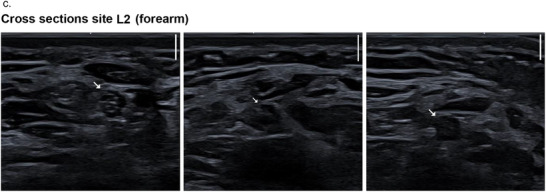

The loss of the fascicular structure and the lack of demarcation of the nerves from the surrounding connective tissue were most noticeable during the dynamic US examinations. These structural changes were more pronounced at Location 2 (forearm) and Location 3 (elbow) than at Location 1 (wrist). Figure 4c consists of exemplary images illustrating these sonomorphological findings.

For each participant in the main study group, the ultrasound images from which all measurements were taken were collected and compiled in Appendix D of Supporting Information. These repeated measurements showed that HRUS is a viable method for monitoring nerve growth in children with SMA.

### 3.2 Evaluation of the CSA

The CSAs were smaller in most children with SMA than in the controls, as shown in the scatterplots in Figure 2. However, additional graphical evaluations showed that the increase in CSA with age in both groups can be described by a logarithmic curve.

#### 3.2.1 Linear Regression Models

The results of the linear regression models, as shown in Table 3, demonstrate that while adjusting for log age, children with SMA had a smaller mean CSA than controls. On the left side, the mean difference was − 0.96 mm^2^ (95% CI: − 1.35 to − 0.56) for Site 1, − 1.02 mm^2^ (95% CI: − 1.35 to − 0.69) for Site 2, and − 0.90 mm^2^ (95% CI: − 1.27 to − 0.54) for Site 3. On the right side, the mean difference was − 0.82 mm^2^ (95% CI: − 1.22 to − 0.41) for Site 1, − 0.74 mm^2^ (95% CI: − 1.04 to − 0.45) for Site 2, and − 0.70 mm^2^ (95% CI: − 1.14 to − 0.25) for Site 3. All mean differences were statistically significant with *p* values < 0.008.

#### 3.2.2 Further Graphical Evaluations

**FIGURE 5:**
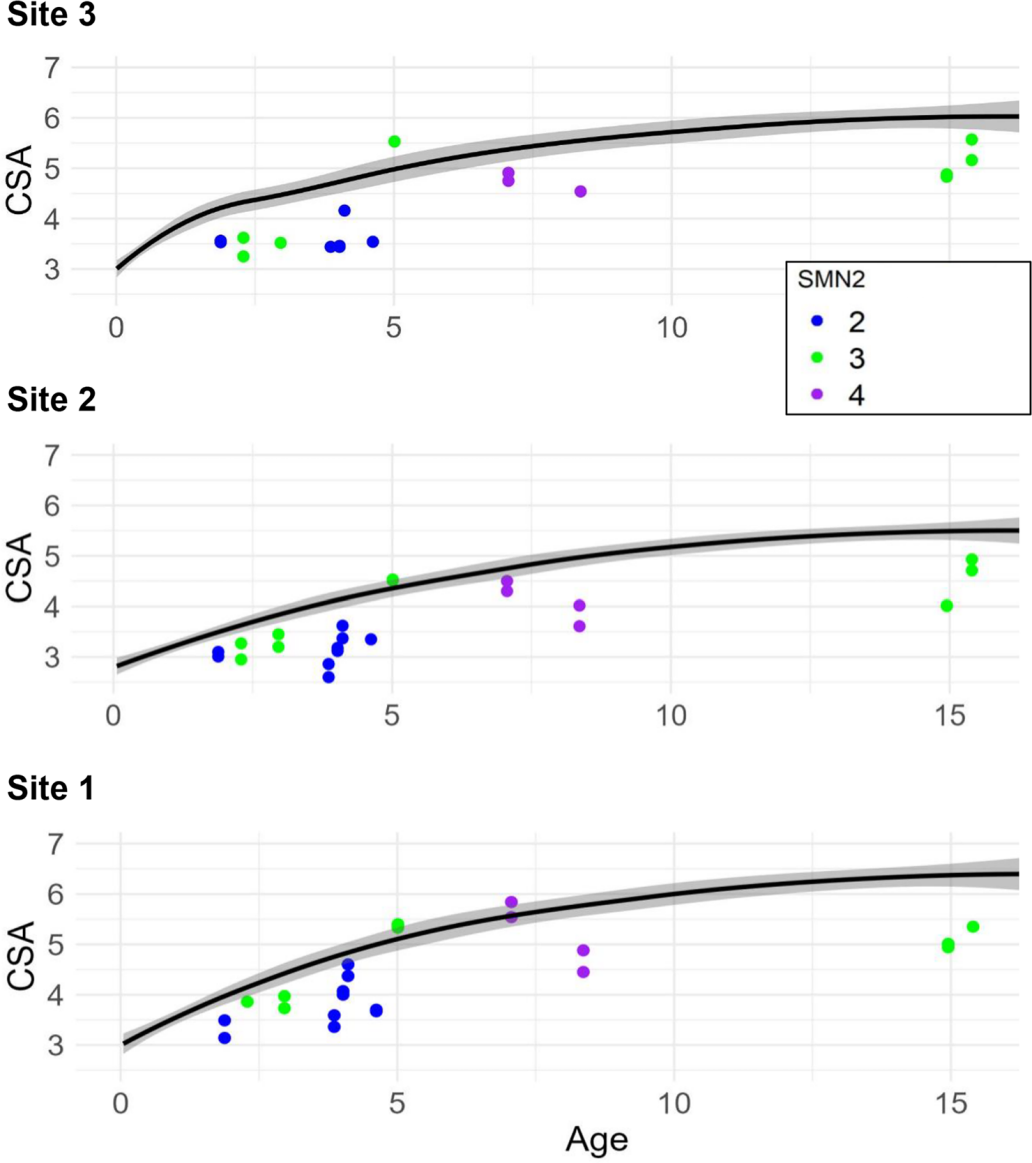
Visual of the SMN2 copy number and the CSA in the SMA group. The plot is similar to that in Figure 4 (Site 2) but supplemented by a color coding of the SMN2 copy number (2 copies: blue, 3 copies: green, 4 copies: purple). SMN2: survival of the motor neuron 2 gene.

In additional graphical evaluations, an association was established between the number of SMN2 copies and nerve CSA, as shown in Figure 5. Thus, participants with only two SMN2 copies often showed a clearer downward deviation from the CSA than the participants with three or more copies.

Furthermore, certain variables, such as weight and head circumference, showed a correlation with the nerve CSA. This correlation can be explained by the fact that all these parameters show an age‐dependent logarithmic growth. However, in contrast to nerve CSA, head circumference did not differ between the SMA and control groups (Appendix A, in the Supporting Information).

## 4 Discussion

This study investigated differences in nerve structure and CSA in children with SMA and healthy controls.

The main finding of this study was that the control population had a higher mean CSA than the SMA population when adjusting for age. Knowing that nerve growth and thus the rate of myelination in normally developing children can be described using a logarithmic model, the growth behavior of the median nerve in the SMA group was investigated graphically (Jenny et al. 2020; Schröder et al. 1988). This showed that, similar to normally developed children, the increase in CSA in children with SMA follows a logarithmic curve.

The second major finding of this study was that the sonographic visualization and delineation of the nerves were noticeably more difficult in most children with SMA than in the controls. To some extent, this can be attributed to the atrophy and fibrotic transformation of the surrounding musculature, as this results in the loss of important anatomical structures that normally provide sonographic guidance. In addition, dynamic examination showed that the fascicular structure of the nerves that resulted in a honeycomb appearance, as described by Peer and Gruber (2013), was barely recognizable in the children with SMA. Given the fact that the visualization of the nerves proved to be particularly difficult in the children with severe clinical manifestations of SMA (markedly atrophied muscles, fibrotic transformation, early onset of symptoms, and few SMN2 copies), an association can be assumed. However, due to the small number of participants and the broad age range a standardized analysis was not realizable.

For a rough comparison between the development of the peripheral nervous system (PNS) and that of the central nervous system (CNS), head circumference in relation to age was analyzed graphically. In contrast to the CSA as a proxy for the development of the PNS, head circumference, as a surrogate for the maturation of the CNS, did not differ between the SMA and control groups (Jenny et al. 2020; Cooke et al. 1977; Koshy et al. 2021).

The key finding of this study, which is the smaller CSA of the median nerve already at the age of 2.5 years, highlights the importance of the SMN protein for the development of the PNS in general and suggests the involvement of other structures besides the motor axons, also including non‐neural tissue.

This statement is in line with recent studies that have investigated the role of the SMN protein and attributed various “housekeeping functions” to it (Chaytow et al. 2018; Faravelli et al. 2023; Detering et al. 2022; Duman et al. 2013).

The argument is strengthened when considering the work of Gesslbauer et al. (2017), which showed that the median nerve at the level of the forearm comprises around 41,000 axons, of which approximately 500 are motor axons. This corresponds to a percentage of just under 1.3%. Therefore, the proportion of CSA loss attributable to motor neuron damage ranges from 5.1% to 7.3% (Appendix B of Supporting Information). Considering that alpha motor neurons are approximately two to three times thicker than sensory neurons (Erlanger and Gasser 1937), this proportion would increase to 10.2% to 21.9%. Consequently, 78.1%–89.8% of the observed CSA reduction can be attributed to more systemic underlying mechanisms. Assuming that only motor axons are pathologically altered in children with SMA, no measurable change in the CSA could be expected.

Furthermore, various electroneuromyographic studies obtained on lower limb nerves and studies in mouse models suggest that in children with SMA Type I, not only the motor but also the sensory nervous system is impaired (Yonekawa et al. 2013; Duman et al. 2013; Pro et al. 2021; Jablonka et al. 2006). This finding is consistent with the observation that children with few SMN2 copies tend to be significantly below the CSA age norms.

In contrast to our findings, Regensburger et al. observed no HRUS abnormalities, except for a reduced number of fascicles in patients with SMA Type I (Regensburger et al. 2020). However, as more than 40% of the patients in our cohort were diagnosed with SMA Type I, this may partly explain the discrepancies between our results and those reported in previous studies. Furthermore, these differences underscore the potential influence of SMA subtypes on sonographic changes and highlight the need for stratified analyses when comparing SMA subtypes.

Finally, our finding of a smaller nerve CSA in patients with SMA is supported by two recent MRI studies that also demonstrated a reduced CSA in patients with SMA (Kollmer et al. 2019; Kollmer et al. 2021). However, these studies were conducted on the lower extremities of adults with SMA Type II or Type III and not in pediatric patients or on the upper extremities.

Thus, this study not only confirms the validity of repeated HRUS nerve measurements in children with SMA but also provides new insights into the early onset of reduced nerve CSA and sonomorphological changes in these patients.

### 4.1 Limitations

The main limitation of this study was the small number of participants in the SMA group. We accounted for this small sample size by providing a more conservative p‐value adjustment, limiting the number of statistical tests performed, and reporting the mean differences and confidence intervals to show the trend that the SMA group had a smaller CSA on average than the control group.

A further limitation is the lack of data for the age group of 0–2 years in the SMA population, which is important because approximately two‐thirds of the growth of the CSA occurs during this time (Jenny et al. 2020).

A third limitation was that the examinations were restricted to the mixed motor and sensory median nerve and to the upper extremity only. The results may deviate if the examinations were made on the lower extremity, more proximal localizations, or a purely sensory nerve.

### 4.2 Conclusion

To summarize, this study shows that in pediatric patients with SMA, nerves have a significantly reduced CSA and an altered sonographic appearance. Furthermore, longitudinal data provided in the Appendix demonstrated that high‐resolution ultrasonography is a viable technique for monitoring nerve growth in children with SMA.

## Author Contributions

J.W. and P.J.B. conceptualized and designed the study. J.W. was responsible for project administration, data curation, validation, and investigation, including ultrasound examinations and all ultrasound measurements. J.W. also performed the visualization and wrote the original draft, as well as the review and editing of the manuscript. N.P.B., L.J., S.M., and P.J.B. supported the ultrasound examinations. Formal analysis was performed by E.W., with contributions from J.W. and P.J.B. to data analysis and interpretation. P.J.B. acquired the funding and supervised the research activity. J.W. and P.J.B. had full access to all study data and took responsibility for data integrity and analytical accuracy. All authors approved the final version of the manuscript.

## Funding

This work was supported by Canon Medical Systems (Tokyo, Japan), the Stiftung OPOS zugunsten von Wahrnehmungsbehinderten, and received intramural funding from the Children's Hospital of Eastern Switzerland.

## Ethics Statement

The study was approved by the local ethics committee of St. Gallen (EKOS approval no. 2019–02200), and all participants' caregivers provided written informed consent before the participants were included in the study.

## Conflicts of Interest

The authors declare no conflicts of interest.

## Supporting information




**Supplementary material**: brb371234‐sup‐0001‐Appendix.docx

## Data Availability

The data that support the findings of this study are available in the Appendix. The data are not publicly available due to privacy or ethical restrictions. Especially to protect vulnerable patient groups (children).

## References

[brb371234-bib-0001] Broser, P. , and J. Lütschg . 2024. “Erworbene Polyneuropathien im Kindesalter.” Neuropaediatrie 23: 110–118.

[brb371234-bib-0002] Calucho, M. , S. Bernal , L. Alías , et al. 2018. “Correlation Between SMA Type and SMN2 Copy Number Revisited: An Analysis of 625 Unrelated Spanish Patients and a Compilation of 2834 Reported Cases.” Neuromuscular Disorders 28, no. 3: 208–215. 10.1016/j.nmd.2018.01.003. 29433793

[brb371234-bib-0003] Chaytow, H. , Y.‐T. Huang , T. H. Gillingwater , and K. M. E. Faller . 2018. “The Role of Survival Motor Neuron Protein (SMN) in Protein Homeostasis.” Cellular and Molecular Life Sciences 75, no. 21: 3877–3894. 10.1007/s00018-018-2849-1. 29872871 PMC6182345

[brb371234-bib-0004] Cooke, R. W. I. , A. Lucas , and P. L. N. Yudkin . 1977. “Pryse‐Davies J. Head Circumference as an Index of Brain Weight in the Fetus and Newborn.” Early Human Development 1, no. 2: 145–149. 10.1016/0378-3782(77)90015-9. 617306

[brb371234-bib-0005] Detering, N. T. , T. Schüning , N. Hensel , and P. Claus . 2022. “The Phospho‐Landscape of the Survival of Motoneuron Protein (SMN) Protein: Relevance for Spinal Muscular Atrophy (SMA).” Cellular and Molecular Life Sciences 79, no. 9: 497. 10.1007/s00018-022-04522-9. 36006469 PMC11071818

[brb371234-bib-0006] Duman, O. , H. Uysal , K. L. Skjei , F. Kizilay , S. Karauzum , and S. Haspolat . 2013. “Sensorimotor Polyneuropathy in Patients With SMA Type‐1: Electroneuromyographic Findings.” Muscle & Nerve 48, no. 1: 117–121. 10.1002/mus.23722. 23629817

[brb371234-bib-0007] Erdos, J. , and C. Wild . 2022. “Mid‐ and Long‐Term (At Least 12 Months) Follow‐Up of Patients With Spinal Muscular Atrophy (SMA) Treated With Nusinersen, Onasemnogene Abeparvovec, Risdiplam or Combination Therapies: A Systematic Review of Real‐World Study Data.” European Journal of Paediatrics Neurology 39: 1–10. 10.1016/j.ejpn.2022.04.006. 35533607

[brb371234-bib-0008] Erlanger, J. , and H. S. Gasser . 1937. Electrical Signs of Nervous Activity. University of Pennsylvania Press.

[brb371234-bib-0009] Faravelli, I. , G. M. Riboldi , P. Rinchetti , and F. Lotti . 2023. “The SMN Complex at the Crossroad Between RNA Metabolism and Neurodegeneration.” International Journal of Molecular Science 24, no. 3: 2247. 10.3390/ijms24032247. PMC9917330 36768569

[brb371234-bib-0010] Gesslbauer, B. , L. A. Hruby , A. D. Roche , D. Farina , R. Blumer , and O. C. Aszmann . 2017. “Axonal Components of Nerves Innervating the Human Arm.” Annals of Neurology 82, no. 3: 396–408. 10.1002/ana.25018. 28833372

[brb371234-bib-0011] Jablonka, S. , K. Karle , B. Sandner , C. Andreassi , K. von Au , and M. Sendtner . 2006. “Distinct and Overlapping Alterations in Motor and Sensory Neurons in a Mouse Model of Spinal Muscular Atrophy.” Human Molecular Genetics 15, no. 3: 511–518. 10.1093/hmg/ddi467. 16396995

[brb371234-bib-0012] Jenny, C. , J. Lütschg , and P. J. Broser . 2020. “Change in Cross‐Sectional Area of the Median Nerve With Age in Neonates, Infants and Children Analyzed by High‐Resolution Ultrasound Imaging.” European Journal of Paediatrics Neurology 29: 137–143. 10.1016/j.ejpn.2020.07.017. 32826155

[brb371234-bib-0013] Kollmer, J. , T. Hilgenfeld , A. Ziegler , et al. 2019. “Quantitative MR Neurography Biomarkers in 5q‐Linked Spinal Muscular Atrophy.” Neurology 93, no. 7: 653–664. 10.1212/WNL.0000000000007945. 31292223

[brb371234-bib-0014] Kollmer, J. , T. Kessler , G. Sam , et al. 2021. “Magnetization Transfer Ratio: A Quantitative Imaging Biomarker for 5q Spinal Muscular Atrophy.” European Journal of Paediatric Neurology 28, no. 1: 331–340. 10.1111/ene.14528. 32918834

[brb371234-bib-0015] Koshy, B. , M. Srinivasan , T. P. Murugan , et al. 2021. “Association Between Head Circumference at Two Years and Second and Fifth Year Cognition.” BMC Pediatrics 21, no. 1: 74. 10.1186/s12887-021-02543-0. 33573614 PMC7876785

[brb371234-bib-0016] Lunn, M. R. , and C. H. Wang . 2008. “Spinal Muscular Atrophy.” Lancet 371, no. 9630: 2120–2133. 10.1016/S0140-6736(08)60921-6. 18572081

[brb371234-bib-0017] Murray, L. M. , L. H. Comley , D. Thomson , et al. 2008. “Selective Vulnerability of Motor Neurons and Dissociation of Pre‐ and Post‐Synaptic Pathology at the Neuromuscular Junction in Mouse Models of Spinal Muscular Atrophy.” Human Molecular Genetics 17, no. 7: 949–962. 10.1093/hmg/ddm367. 18065780

[brb371234-bib-0018] Pearn, J. 1980. “Classification of Spinal Muscular Atrophies.” Lancet 1, no. 8174: 919–922. 10.1016/s0140-6736(80)90847-8. 6103267

[brb371234-bib-0019] Peer, S. , and H. Gruber . 2013. Atlas of Peripheral Nerve Ultrasound. Springer.

[brb371234-bib-0020] Prior, T. W. , M. E. Leach , and E. Finanger . 2000. “Spinal Muscular Atrophy.” In GeneReviews, edited by M. P. Adam , J. Feldman , G. M. Mirzaa , et al. University of Washington. https://www.ncbi.nlm.nih.gov/books/. 20301526

[brb371234-bib-0021] Pro, S. , A. E. Tozzi , A. D'Amico , et al. 2021. “Age‐Related Sensory Neuropathy in Patients With Spinal Muscular Atrophy Type 1.” Muscle & Nerve 64, no. 5: 599–603. 10.1002/mus.27389. 34368972

[brb371234-bib-0022] R Core Team . 2023. R: A Language and Environment for Statistical Computing. R Foundation for Statistical Computing.

[brb371234-bib-0023] Regensburger, A. P. , A. L. Wagner , G. Hanslik , et al. 2020. “Ultra‐High‐Frequency Ultrasound in Patients With Spinal Muscular Atrophy: A Retrospective Feasibility Study.” Muscle & Nerve 61, no. 3: E18–E21. 10.1002/mus.26796. 31884704

[brb371234-bib-0024] Russman, B. S. 2007. “Spinal Muscular Atrophy: Clinical Classification and Disease Heterogeneity.” Journal of Child Neurology 22, no. 8: 946–951. 10.1177/0883073807305673. 17761648

[brb371234-bib-0025] Schröder, J. M. , J. Bohl , and U. von Bardeleben . 1988. “Changes of the Ratio Between Myelin Thickness and Axon Diameter in Human Developing Sural, Femoral, Ulnar, Facial, and Trochlear Nerves.” Acta Neuropathologica 76, no. 5: 471–483. 10.1007/BF00686386. 3188839

[brb371234-bib-0026] Wadman, R. I. , W. L. van der Pol , W. M. J. Bosboom , et al. 2019. “Drug Treatment for Spinal Muscular Atrophy Type I.” Cochrane Database of Systematic Reviews 12, no. 12: Cd006281. 10.1002/14651858.CD006281.pub5. 31825542 PMC6905354

[brb371234-bib-0027] Wickham, H. 2016. ggplot2: Elegant Graphics for Data Analysis. 2nd ed. Springer.

[brb371234-bib-0028] Yonekawa, T. , H. Komaki , Y. Saito , K. Sugai , and M. Sasaki . 2013. “Peripheral Nerve Abnormalities in Pediatric Patients With Spinal Muscular Atrophy.” Brain & Development 35, no. 2: 165–171. 10.1016/j.braindev.2012.03.009. 22512990

