# Supplementary material for: Examination of the Peripheral Nervous System in Children With Spinal Muscular Atrophy: A High‐Resolution Ultrasonographic Study
Source: Brain Behav. 2026 Feb 5;16(2):e71234. doi: 10.1002/brb3.71234 (PMC12876041; doi:10.1002/brb3.71234)
Supplement: Supplementary file 1 — Supplementary material: brb371234‐sup‐0001‐Appendix.docx [file BRB3-16-e71234-s001.docx]

**SMA Ultrasonographic Study**

Janina Wurster, Dr. Erin West, Sandro Meier, Noé Phillip Bürke, Lynn Jansen, PD Dr. Med. Philip Julian Broser

2026-01-14

**Contents**

**Appendix A, Fig. A.1:** Graphical analysis of head circumferences

**Appendix B, Table B.1:** Percentage of CSA reduction attributable to motor axon damage

**Appendix C, Table C.1:** Measurement results from all sonographic examinations

**Appendix D, Fig. D.1:** Compilation of the ultrasound images of the participants with SMA

**Appendix A, Fig. A.1:** Graphical analysis of head circumferences

Scatterplot of the head circumferences in cm and ages in years. The control group is represented by the trend line in red with the confidence interval in gray. The head circumferences of the SMA group are represented by blue dots.

**
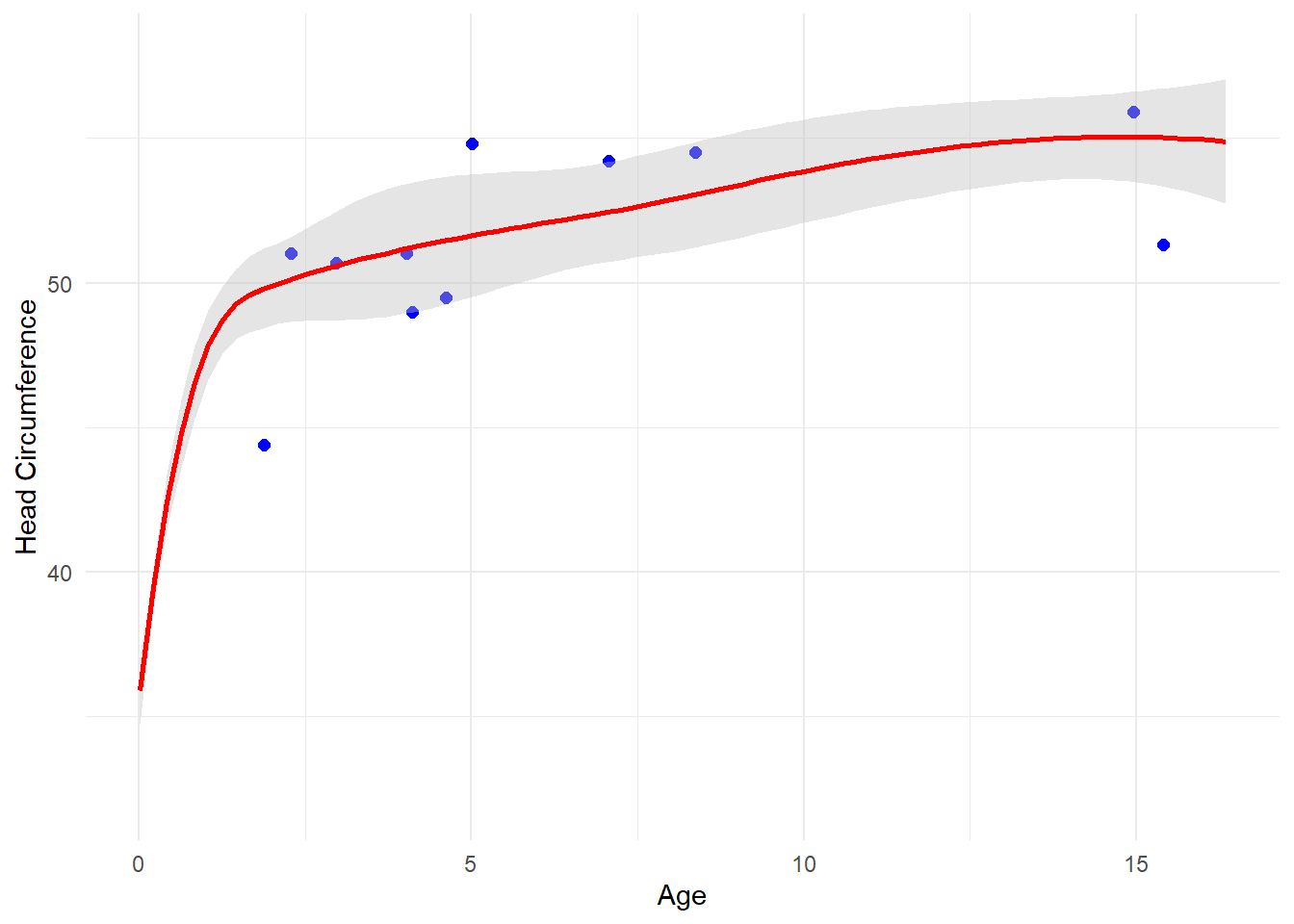
**

**Appendix B, Table B.1:** Percentage of CSA reduction attributable to motor axon damage

|  | **Left Arm** | |  | **Right Arm** | |  |
| --- | --- | --- | --- | --- | --- | --- |
|  | **Mean area (mm^2^)** | **Mean difference (mm^2^)** | **CSA decrease due to motor axon loss (in %)** | **Mean area (mm^2^)** | **Mean difference (mm^2^)** | **CSA decrease due to motor axon loss (in %)** |
| **Site 1** | 4.52 | -0.96 | 6.1 | 4.27 | -0.82 | 6.8 |
| **Site 2** | 4.01 | -1.02 | 5.1 | 3.74 | -0.74 | 6.6 |
| **Site 3** | 4.26 | -0.90 | 6.2 | 3.94 | -0.70 | 7.3 |

Shown is the percentage of median nerve CSA reduction attributable to motor axon damage at sites 1 to 3 for both arms. Calculations are based on data from Gesslbauer et al. (22), indicating that motor axons account for approximately 1.3% of the axons in the median nerve. The maximal CSA reduction due to motor axon damage was therefore assumed to be 1.3% of the mean CSA and expressed relative to the observed mean CSA difference.

**Appendix C, Table C.1:** Measurement results from all sonographic examinations

| **Demographic data and body mass** | | | | | | | **CSA and circumference of the median nerve** | | | | | | | | | | | |
| --- | --- | --- | --- | --- | --- | --- | --- | --- | --- | --- | --- | --- | --- | --- | --- | --- | --- | --- |
|  |  |  |  |  |  |  | **Left arm** | | | | | | **Right arm** | | | | | |
|  |  |  |  |  |  |  | **Site 1** | | **Site 2** | | **Site 3** | | **Site 1** | | **Site 2** | | **Site 3** | |
| **ID** | **Group** | **Age [years]** | **Sex** | **Weight [kg]** | **Height [cm]** | **Headcirc [cm]** | **L1 area** | **L1 circ.** | **L2 area** | **L2 circ.** | **L3 area** | **L3 circ.** | **R1 area** | **R1 circ.** | **R2 area** | **R2 circ.** | **R3 area** | **R3 circ.** |
| 1 | SMA | 15.4 | f | 37.0 | 154.7 | 51.3 | 5.35 | 11.93 | 4.93 | 9.13 | 5.16 | 8.43 |  |  | 4.71 | 8.20 | 5.57 | 9.91 |
| 2 | SMA | 7.1 | f | 19.0 | 113.5 | 54.2 | 5.84 | 10.77 | 4.50 | 7.98 | 4.91 | 8.58 | 5.54 | 10.28 | 4.30 | 7.68 | 4.75 | 8.57 |
| 3 | SMA | 3.0 | m | 13.6 | 91.2 | 50.7 | 3.73 | 10.50 | 3.20 | 6.71 |  |  | 3.97 | 9.63 | 3.45 | 6.90 | 3.52 | 7.60 |
| 4 | SMA | 4.1 | f | 17.9 | 104.0 | 49.0 | 4.37 | 10.31 | 3.37 | 6.89 | 4.16 | 8.07 | 4.60 | 9.51 | 3.62 | 7.47 |  |  |
| 5 | SMA | 15.0 | f | 31.3 | 161.0 | 55.9 | 4.94 | 9.73 | 4.01 | 7.54 | 4.88 | 8.44 | 5.01 | 12.42 | 4.02 | 7.88 | 4.83 | 8.69 |
| 6 | SMA | 8.4 | f | 29.4 | 122.5 | 54.5 | 4.88 | 10.31 | 4.02 | 7.64 | 4.54 | 8.11 | 4.45 | 10.73 | 3.61 | 7.21 |  |  |
| 7 | SMA | 2.3 | m |  |  | 51.0 |  |  | 2.95 | 6.59 | 3.25 | 7.50 | 3.86 | 9.28 | 3.27 | 7.13 | 3.62 | 7.63 |
| 8 | SMA | 4.6 | f | 15.6 | 95.0 | 49.5 | 3.70 | 9.64 |  |  |  |  | 3.67 | 10.33 | 3.35 | 7.01 | 3.54 | 7.29 |
| 9 | SMA | 1.9 | f |  |  | 44.4 | 3.14 | 8.63 | 3.10 | 6.81 | 3.53 | 7.41 | 3.49 | 10.53 | 3.01 | 6.67 | 3.56 | 7.10 |
| 10 | SMA | 5.0 | m | 23.3 | 103.5 | 54.8 | 5.33 | 11.16 | 4.51 | 8.51 | 5.53 | 8.96 | 5.40 | 12.25 | 4.53 | 8.25 |  |  |
| 11 | SMA | 3.9 | f | 13.0 | 96.0 |  | 3.36 | 9.97 | 2.60 | 6.18 | 3.44 | 7.70 | 3.59 | 9.36 | 2.86 | 6.55 |  |  |
| 12 | SMA | 4.0 | m | 11.8 | 95.0 | 51.0 | 4.07 | 8.99 | 3.12 | 6.53 | 3.44 | 6.84 | 4.00 | 8.49 | 3.18 | 6.57 | 3.46 | 7.06 |
| 13 | Control | 7.0 | f | 22.7 | 119.0 |  |  |  |  |  |  |  | 6.76 | 11.98 | 5.56 | 8.66 |  |  |
| 14 | Control | 4.4 | m | 14.0 | 99.0 |  |  |  |  |  |  |  | 4.48 | 10.49 |  |  |  |  |
| 15 | Control | 14.9 | f | 56.0 | 164.3 |  |  |  |  |  |  |  |  |  | 4.55 | 8.35 |  |  |
| 16 | Control | 12.9 | f | 32.8 | 138.3 |  |  |  |  |  |  |  | 5.30 | 11.52 | 4.56 | 8.35 |  |  |
| 17 | Control | 13.7 | f | 61.3 | 166.0 |  |  |  |  |  |  |  | 4.81 | 9.97 | 4.50 | 8.30 |  |  |
| 18 | Control | 9.6 | f | 55.0 | 146.3 |  |  |  |  |  |  |  | 5.63 | 12.62 | 4.80 | 8.55 |  |  |
| 19 | Control | 17.2 | f | 68.7 | 169.8 |  | 5.75 | 10.55 |  |  |  |  |  |  |  |  |  |  |
| 20 | Control | 8.6 | f | 25.6 | 128.1 | 51.0 |  |  |  |  |  |  |  |  | 4.70 | 8.97 |  |  |
| 21 | Control | 6.1 | f | 21.5 | 116.7 |  | 5.18 | 11.18 | 4.13 | 7.51 |  |  |  |  |  |  |  |  |
| 22 | Control | 14.0 | f | 69.5 | 162.5 |  | 6.92 | 12.05 | 5.79 | 9.43 |  |  |  |  |  |  |  |  |
| 23 | Control | 11.3 | f | 40.0 | 149.3 |  |  |  |  |  |  |  | 5.79 | 10.60 | 5.51 | 9.26 |  |  |
| 24 | Control | 6.0 | m | 16.2 | 111.0 |  | 4.73 | 10.27 | 3.89 | 7.51 |  |  |  |  |  |  |  |  |
| 25 | Control | 10.4 | m | 44.0 | 158.0 |  |  |  |  |  |  |  |  |  | 6.09 | 9.04 |  |  |
| 26 | Control | 14.7 | f | 53.5 | 163.0 |  | 8.17 | 14.16 |  |  |  |  |  |  |  |  |  |  |
| 27 | Control | 12.2 | m | 63.7 | 167.1 |  | 6.87 | 13.44 | 5.20 | 9.46 |  |  |  |  |  |  |  |  |
| 28 | Control | 5.3 | f | 20.8 | 113.5 |  |  |  |  |  |  |  | 5.21 | 11.05 | 3.87 | 8.29 |  |  |
| 29 | Control | 14.9 | f | 53.7 | 169.5 | 56.5 | 6.93 | 13.04 | 6.64 | 9.81 |  |  |  |  |  |  |  |  |
| 30 | Control | 11.7 | f | 33.4 | 146.5 |  |  |  |  |  |  |  |  |  | 4.41 | 8.66 |  |  |
| 31 | Control | 9.8 | f | 28.2 | 132.5 |  |  |  |  |  |  |  | 6.50 | 13.36 | 4.95 | 9.33 |  |  |
| 32 | Control | 9.3 | m | 52.5 | 148.0 | 55.0 |  |  | 5.17 | 8.65 |  |  |  |  |  |  |  |  |
| 33 | Control | 8.1 | f | 33.0 | 128.6 | 52.5 | 5.26 | 9.87 | 5.07 | 8.97 | 6.16 | 10.53 |  |  |  |  |  |  |
| 34 | Control | 10.8 | f | 48.0 | 149.3 |  | 5.43 | 11.65 | 6.05 | 9.45 | 6.39 | 9.85 |  |  |  |  |  |  |
| 35 | Control | 14.9 | f | 60.8 | 166.0 |  | 6.27 | 13.58 | 5.63 | 9.48 | 6.43 | 10.20 | 7.01 | 13.75 |  |  |  |  |
| 36 | Control | 7.2 | m | 24.2 | 122.3 |  |  |  |  |  |  |  | 6.46 | 12.91 | 4.71 | 8.68 |  |  |
| 37 | Control | 14.1 | f | 78.0 | 166.0 | 57.5 | 6.65 | 12.49 | 6.08 | 10.03 |  |  |  |  |  |  |  |  |
| 38 | Control | 7.0 | f | 22.0 | 126.0 |  | 6.20 | 10.56 | 5.46 | 8.58 |  |  |  |  |  |  |  |  |
| 39 | Control | 8.9 | f | 21.6 | 127.0 |  |  |  |  |  |  |  | 5.83 | 12.39 | 4.94 | 8.58 |  |  |
| 40 | Control | 16.2 | m | 78.0 | 180.5 |  | 6.81 | 11.68 | 6.25 | 9.64 |  |  |  |  |  |  |  |  |
| 41 | Control | 5.7 | m | 16.5 | 109.5 |  |  |  |  |  |  |  | 5.88 | 12.19 | 4.55 | 8.33 |  |  |
| 42 | Control | 15.7 | m | 57.6 | 176.2 |  |  |  | 5.17 | 8.33 | 6.04 | 10.01 |  |  | 4.77 | 8.15 | 6.00 | 10.12 |
| 43 | Control | 12.3 | f | 50.0 | 156.0 |  |  |  |  |  |  |  |  |  | 5.14 | 8.96 | 6.25 | 9.98 |
| 44 | Control | 6.8 | f | 22.1 | 121.5 |  | 5.70 | 13.75 | 5.60 | 9.68 | 6.02 | 12.28 |  |  |  |  |  |  |
| 45 | Control | 14.2 | m | 63.0 | 167.0 |  |  |  | 5.45 | 8.58 | 5.48 | 9.65 |  |  |  |  |  |  |
| 46 | Control | 13.9 | m | 66.1 | 172.6 |  | 5.36 | 11.63 | 5.48 | 8.94 |  |  |  |  |  |  |  |  |
| 47 | Control | 5.4 | m | 21.0 | 106.0 |  | 5.49 | 12.03 | 5.12 | 9.15 | 5.65 | 9.64 |  |  |  |  |  |  |
| 48 | Control | 8.0 | m | 27.7 | 129.0 |  |  |  |  |  |  |  | 4.32 | 10.20 | 4.10 | 8.48 | 4.64 | 8.50 |
| 49 | Control | 13.3 | f | 50.7 | 161.2 |  |  |  |  |  |  |  | 6.69 | 13.80 | 5.33 | 9.22 |  |  |
| 50 | Control | 14.7 | f | 47.7 | 159.8 |  | 6.49 | 12.47 | 4.97 | 8.61 |  |  |  |  |  |  |  |  |
| 51 | Control | 15.8 | f |  |  | 53.8 | 5.97 | 11.40 | 5.45 | 8.91 | 5.74 | 9.32 |  |  |  |  |  |  |
| 52 | Control | 13.4 | f | 50.0 | 159.0 |  |  |  |  |  |  |  | 7.00 | 12.10 | 5.64 | 8.84 | 6.13 | 9.60 |
| 53 | Control | 16.0 | m |  |  |  | 7.10 | 13.98 | 5.43 | 8.81 |  |  |  |  |  |  |  |  |
| 54 | Control | 10.9 | f |  |  |  | 6.44 | 12.35 | 6.22 | 9.38 | 6.23 | 10.88 |  |  |  |  |  |  |
| 55 | Control | 11.2 | m | 47.5 | 155.0 |  | 6.40 | 11.77 | 5.50 | 9.21 | 5.54 | 9.64 |  |  | 5.79 | 8.87 | 5.24 | 8.65 |
| 56 | Control | 1.3 | m | 13.4 | 86.5 | 52.0 | 4.13 | 9.92 | 3.07 | 7.46 | 3.43 | 8.66 |  |  |  |  | 4.21 | 7.99 |
| 57 | Control | 2.3 | m | 12.4 | 91.0 | 48.0 | 4.78 | 10.10 | 4.02 | 7.64 | 4.51 | 10.30 |  |  |  |  |  |  |
| 58 | Control | 2.0 | m | 13.7 | 87.9 | 50.3 | 4.05 | 11.37 | 3.81 | 7.22 |  |  | 4.28 | 13.38 | 3.70 | 7.60 |  |  |
| 59 | Control | 1.5 | m | 11.0 |  | 47.5 |  |  | 3.32 | 7.26 | 4.53 | 10.15 | 3.53 | 9.62 | 3.10 | 6.96 | 4.36 | 8.89 |
| 60 | Control | 1.2 | m | 11.2 | 77.0 | 46.5 | 3.51 | 8.46 | 2.98 | 6.84 | 3.35 | 6.92 |  |  |  |  |  |  |
| 61 | Control | 2.8 | f | 12.9 | 92.2 | 51.0 | 3.77 | 9.70 | 3.69 | 7.36 | 4.65 | 9.10 |  |  |  |  |  |  |
| 62 | Control | 0.1 | m | 3.14 | 46.5 | 35.0 | 2.10 | 9.20 | 2.16 | 5.79 | 2.96 | 6.42 | 2.43 | 9.22 | 2.30 | 6.34 | 3.14 | 6.83 |
| 63 | Control | 0.9 | m | 10.8 | 72.0 | 48.0 | 3.51 | 8.45 |  |  |  |  | 4.24 | 10.04 | 3.09 | 7.19 | 3.22 | 7.38 |
| 64 | Control | 0.9 | f | 10.3 | 75.0 | 47.5 |  |  |  |  |  |  | 4.18 | 10.22 | 3.51 | 7.18 | 4.03 | 7.81 |
| 65 | Control | 0.5 | f | 6.84 | 68.0 | 44.0 | 3.41 | 8.45 | 2.90 | 6.53 | 3.48 | 7.41 | 3.70 | 9.23 | 3.54 | 7.15 | 3.69 | 7.49 |
| 66 | Control | 0.2 | m | 5.65 | 58.0 | 41.0 |  |  | 2.82 | 7.09 | 3.98 | 7.95 | 3.16 | 8.83 | 2.68 | 6.73 | 3.23 | 6.69 |
| 67 | Control | 0.3 | f | 5.86 | 61.5 | 41.5 | 2.50 | 8.97 | 2.85 | 6.63 | 3.40 | 8.28 | 2.75 | 9.50 | 3.01 | 7.34 | 2.85 | 6.70 |
| 68 | Control | 0.9 | m | 11.1 | 76.0 | 44.2 | 4.14 | 10.77 | 3.35 | 7.01 | 4.24 | 7.52 | 4.10 | 10.65 | 3.39 | 6.77 | 3.36 | 7.09 |
| 69 | Control | 0.0 | m | 3.74 | 50.0 | 38.0 |  |  |  |  | 2.57 | 6.33 |  |  | 2.01 | 5.20 | 3.31 | 6.82 |
| 70 | Control | 0.1 | f | 2.63 | 47.3 | 33.5 | 2.58 | 10.17 | 2.87 | 6.33 | 2.80 | 6.64 | 2.48 | 6.88 | 2.40 | 6.14 | 2.93 | 6.87 |
| 71 | Control | 0.2 | m | 4.58 |  |  | 1.74 | 5.67 | 2.67 | 6.48 |  |  | 2.54 | 7.77 | 2.26 | 6.39 | 2.82 | 6.70 |
| 72 | Control | 0.0 | m | 3.28 | 51.5 | 36.0 | 1.39 | 5.51 | 1.74 | 4.99 | 2.76 | 6.43 | 1.40 | 5.65 | 1.48 | 4.48 | 2.16 | 6.37 |
| 73 | Control | 0.4 | m | 8.10 | 70.0 | 44.0 | 3.22 | 9.37 | 3.01 | 6.45 | 3.92 | 7.69 | 3.36 | 8.71 | 2.98 | 6.96 | 3.92 | 7.72 |
| 74 | Control | 0.1 | m | 4.19 | 52.0 | 37.4 |  |  |  |  |  |  | 2.34 | 7.34 | 2.11 | 5.45 | 2.33 | 6.20 |
| 75 | Control | 0.2 | f | 6.46 | 59.5 | 39.0 | 3.62 | 10.57 | 3.11 | 6.69 | 3.21 | 7.08 | 3.36 | 9.89 | 3.26 | 6.64 | 3.58 | 8.12 |
| 76 | Control | 0.1 | f | 4.20 | 56.0 | 37.8 |  |  |  |  |  |  | 2.74 | 9.19 | 2.92 | 6.30 | 3.59 | 7.42 |
| 77 | Control | 0.0 | f | 4.39 | 56.0 | 37.0 |  |  |  |  |  |  | 3.42 | 10.68 | 2.44 | 6.63 | 3.82 | 9.11 |
| 78 | Control | 0.1 | m | 4.30 | 54.6 | 38.0 | 2.33 | 8.61 | 2.29 | 5.89 | 2.64 | 7.38 |  |  |  |  |  |  |
| 79 | Control | 0.2 | m | 5.04 | 60.5 | 39.0 | 3.12 | 10.85 | 3.11 | 6.72 | 3.35 | 9.31 | 3.14 | 9.82 | 3.08 | 6.49 | 3.48 | 8.04 |
| 80 | Control | 0.1 | m | 5.52 | 59.0 | 39.5 | 1.97 | 8.36 | 1.84 | 5.33 | 2.11 | 5.31 |  |  |  |  |  |  |
| 81 | Control | 0.3 | f | 5.73 | 65.0 | 40.0 |  |  |  |  |  |  | 3.50 | 9.17 | 3.06 | 6.55 |  |  |
| 82 | Control | 0.1 | f | 3.54 | 53.2 | 35.0 | 2.51 | 7.07 | 2.05 | 6.10 | 3.23 | 6.70 |  |  |  |  |  |  |
| 83 | Control | 0.2 | m | 4.96 | 59.0 | 38.0 | 3.25 | 8.89 | 2.55 | 5.89 | 3.12 | 7.25 | 3.25 | 8.35 | 2.81 | 7.39 | 3.25 | 7.74 |
| 84 | Control | 0.3 | m | 6.12 | 62.0 | 42.0 | 3.33 | 9.40 | 2.75 | 6.81 | 3.28 | 6.67 | 3.04 | 8.72 | 2.48 | 6.04 | 2.70 | 6.71 |
| 85 | Control | 0.8 | m | 9.57 | 73.0 | 47.0 | 3.34 | 9.69 | 3.07 | 6.67 |  |  | 3.43 | 8.96 | 3.04 | 6.59 |  |  |
| 86 | Control | 0.1 | f | 4.06 | 55.0 | 38.0 | 2.39 | 7.19 | 1.92 | 5.39 | 2.91 | 6.57 | 1.95 | 6.64 | 2.06 | 5.87 | 1.96 | 6.64 |
| 87 | Control | 0.6 | f | 8.40 | 70.5 | 44.5 | 3.20 | 8.60 | 2.87 | 6.65 | 3.50 | 7.27 | 3.08 | 9.09 | 2.89 | 6.43 | 3.90 | 8.71 |
| 88 | Control | 0.3 | f | 6.12 | 60.0 | 39.9 | 2.07 | 7.65 | 2.19 | 5.57 | 2.78 | 6.88 | 2.55 | 8.08 | 2.65 | 6.08 | 2.64 | 8.39 |
| 89 | Control | 0.1 | m | 5.70 | 56.7 | 40.2 |  |  | 2.68 | 6.43 | 2.89 | 7.33 |  |  | 2.71 | 6.45 | 3.09 | 8.14 |
| 90 | Control | 0.3 | f | 5.06 | 58.5 | 40.5 | 2.81 | 7.67 | 2.68 | 6.61 | 3.09 | 6.78 | 2.99 | 9.42 | 2.20 | 6.96 | 2.79 | 6.84 |
| 91 | Control | 3.5 | m | 14.8 | 95.0 |  | 4.35 | 10.03 | 3.62 | 7.09 | 4.19 | 7.98 |  |  |  |  |  |  |
| 92 | Control | 3.8 | m | 17.0 | 104.3 |  |  |  |  |  |  |  | 4.42 | 10.60 | 4.04 | 7.67 | 4.21 | 7.69 |
| 93 | Control | 3.4 | m | 16.0 | 100.0 |  |  |  |  |  |  |  | 4.33 | 12.55 | 3.92 | 7.54 | 4.41 | 7.72 |
| 94 | Control | 3.0 | f | 12.6 | 84.0 |  | 4.61 | 11.10 | 3.79 | 7.76 | 4.38 | 7.81 | 4.61 | 11.25 | 3.92 | 7.17 | 4.13 | 7.71 |
| 95 | Control | 1.0 | m |  | 77.0 |  |  |  |  |  |  |  | 3.43 | 9.30 | 2.91 | 6.81 | 3.42 | 7.09 |
| 96 | Control | 7.4 | m | 30.7 | 133.0 |  |  |  |  |  |  |  | 4.87 | 11.85 | 4.08 | 7.46 | 4.65 | 8.03 |
| 97 | Control | 1.7 | m | 11.1 | 80.0 |  | 4.04 | 10.48 | 3.47 | 7.29 | 4.26 | 8.25 | 4.16 | 10.37 | 3.69 | 7.67 | 4.51 | 7.85 |
| 98 | Control | 1.4 | m | 12.5 | 87.0 | 49.2 | 3.75 | 10.46 | 3.54 | 7.26 | 4.22 | 8.5 |  |  |  |  |  |  |
| 99 | Control | 2.4 | f | 13.9 | 92.0 | 49.0 | 3.85 | 10.29 | 3.81 | 7.72 | 4.45 | 8.13 | 4.00 | 10.68 | 3.53 | 7.06 | 4.24 | 8.50 |
| 100 | Control | 2.4 | m | 13.0 | 90.0 |  |  |  |  |  |  |  | 3.84 | 9.77 | 3.28 | 7.24 | 3.96 | 7.56 |
| 101 | Control | 10.9 | m | 39.8 | 147.0 |  | 5.97 | 11.35 | 4.94 | 8.87 | 5.71 | 9.69 | 6.35 | 12.77 | 5.06 | 8.48 | 5.22 | 9.53 |
| 102 | Control | 4.5 | f | 18.7 | 107.5 |  | 4.74 | 11.23 | 4.44 | 8.21 | 5.14 | 9.10 |  |  |  |  |  |  |
| 103 | Control | 6.7 | m | 26.3 | 126.0 |  |  |  |  |  |  |  | 5.44 | 13.28 | 4.37 | 7.82 | 5.52 | 8.91 |
| 104 | Control | 2.9 | m | 15.1 | 94.0 |  | 4.54 | 10.17 | 3.85 | 7.41 | 4.79 | 8.65 |  |  |  |  | 4.73 | 8.38 |
| 105 | Control | 2.3 | f | 12.2 | 93.0 |  | 4.07 | 11.05 | 3.43 | 7.15 | 3.84 | 7.85 | 4.45 | 9.80 | 3.79 | 7.68 | 4.06 | 8.27 |
| 106 | Control | 4.4 | m | 18.1 | 110.0 | 53.0 |  |  |  |  |  |  | 5.15 | 12.00 | 4.26 | 7.71 | 4.47 | 8.65 |
| 107 | Control | 16.4 | f | 58.8 | 166.0 | 53.5 | 6.34 | 11.63 | 5.29 | 8.36 | 6.14 | 9.92 | 6.74 | 12.21 | 5.86 | 8.95 | 5.99 | 9.48 |
| 108 | Control | 16.3 | f | 53.0 | 170.0 |  | 6.92 | 12.51 | 5.87 | 9.50 | 6.14 | 9.61 |  |  |  |  |  |  |
| 109 | Control | 4.7 | m | 24.9 | 110.0 |  | 4.90 | 11.28 | 4.32 | 8.52 | 4.85 | 9.23 |  |  |  |  |  |  |

Headcirc: head circumference, area: CSA, circ: circumference

**Appendix D, Fig. D.1:** Compilation of the ultrasound images of the participants with SMA

Following pages

| **ID: 5** | | | | |
| --- | --- | --- | --- | --- |
| Site | Examination 1 | Examination 2 | Examination 3 | |
| L1 | 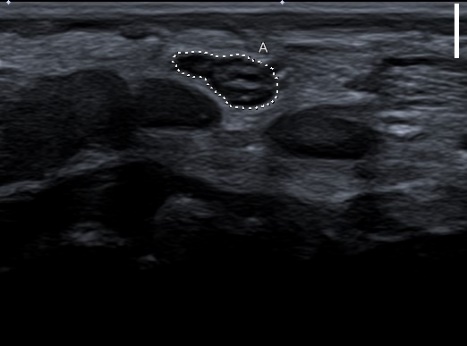 | 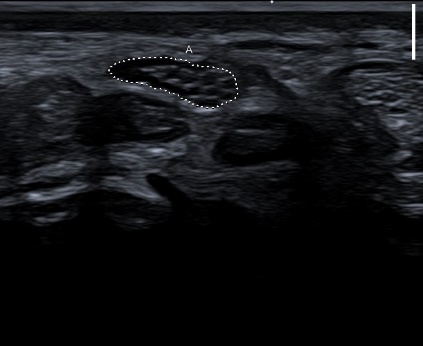 | 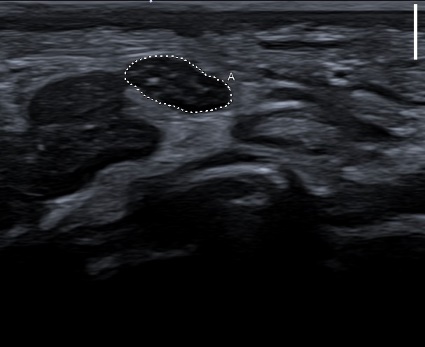 | |
| L2 | 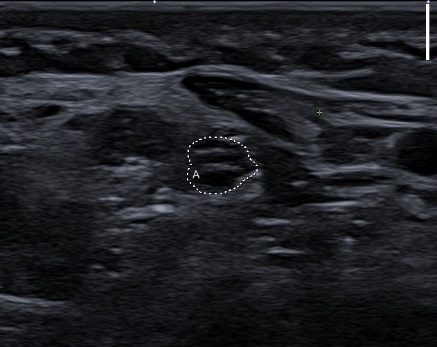 | 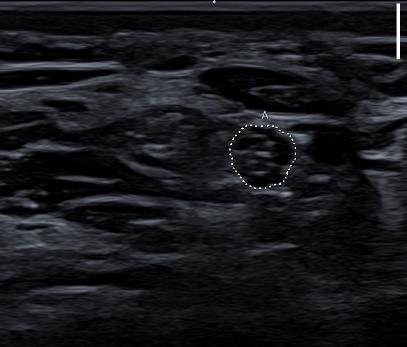 | 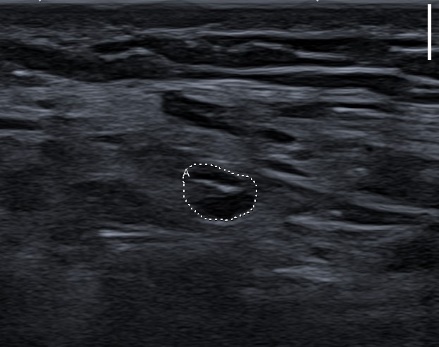 | |
| L3 | 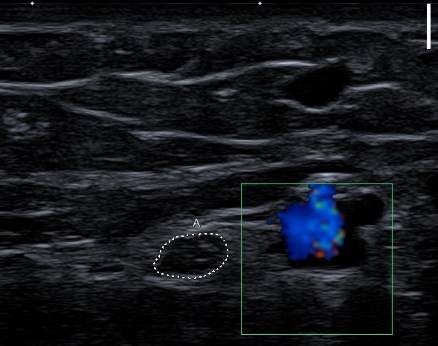 | 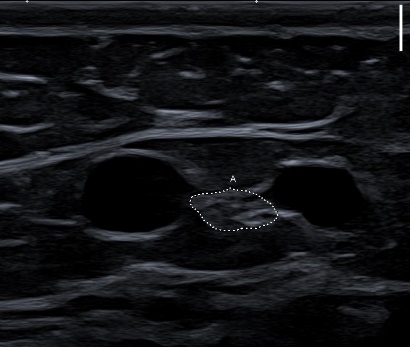 | 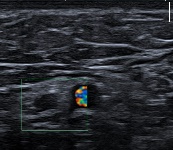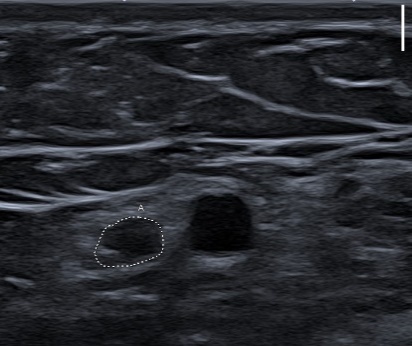 | |
| R1 | 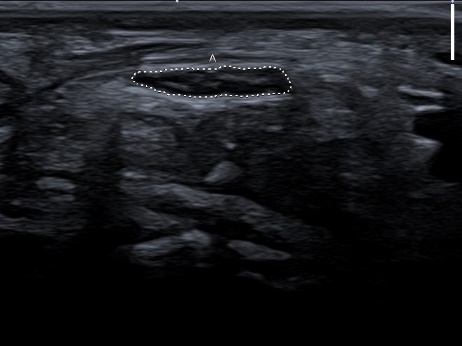 | 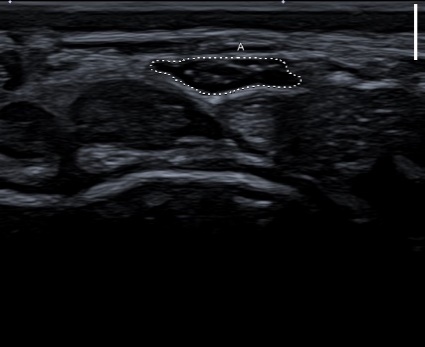 | 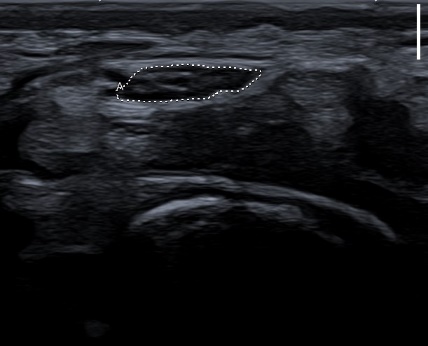 | |
| R2 | 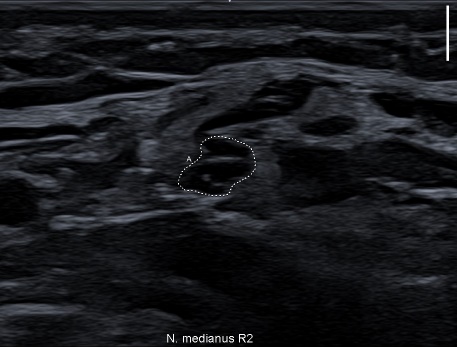 | 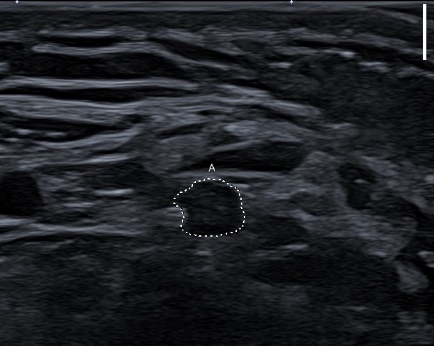 | 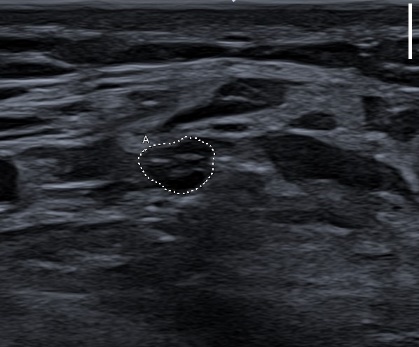 | |
| R3 | 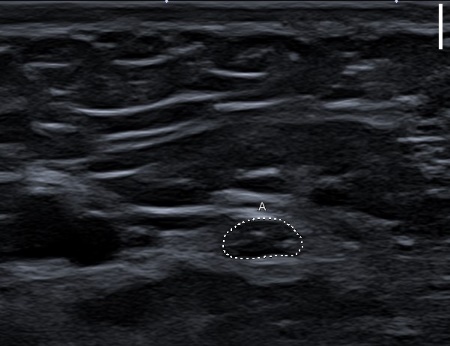 | 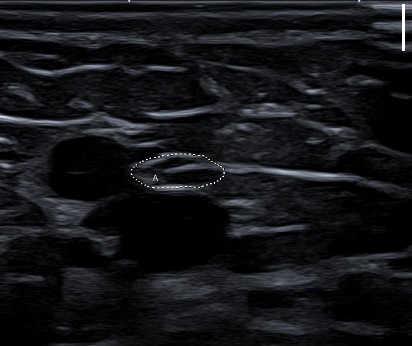 | 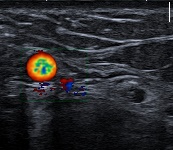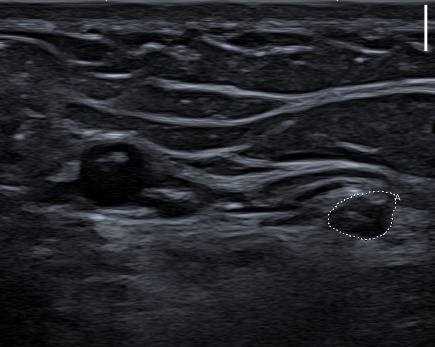 | |
| Longitudinal section | | | |  |
| L2 |  | 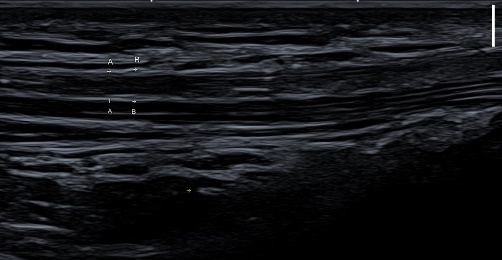 | 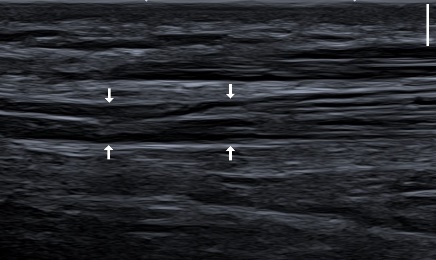 | |
| R2 |  | 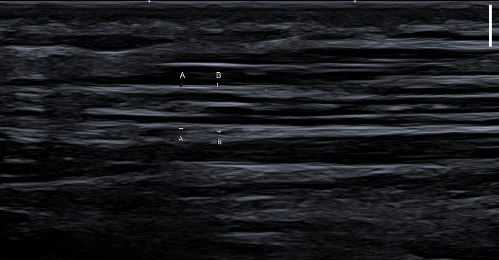 |  | |
| 33 MHz probe | | | | |
| L1 |  |  | 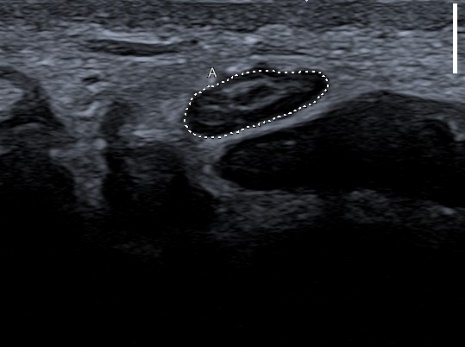 | |
| R1 |  |  | 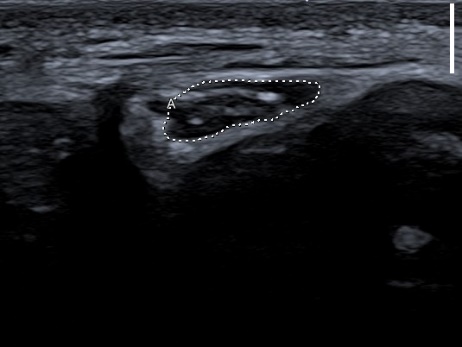 | |

| **ID: 2** | | | |
| --- | --- | --- | --- |
| Site | Examination 1 | Examination 2 | Examination 3 |
| L1 | 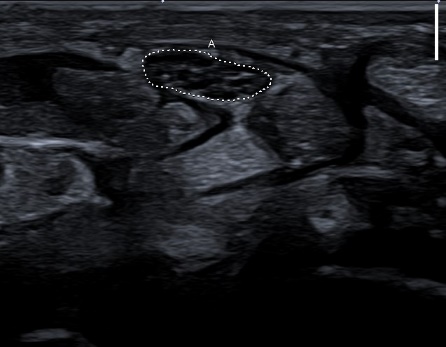 |  | 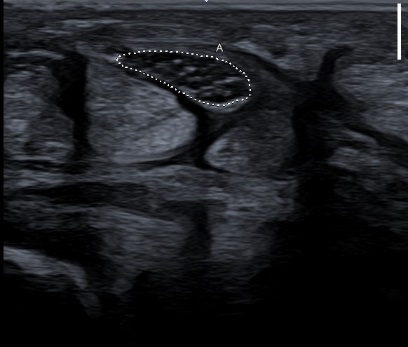 |
| L2 | 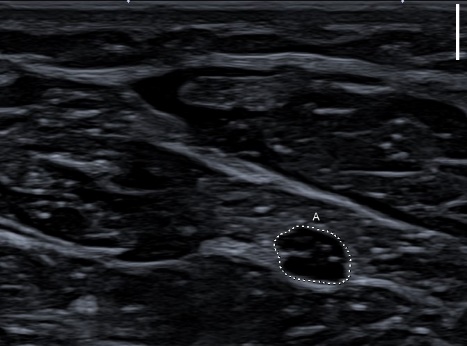 | 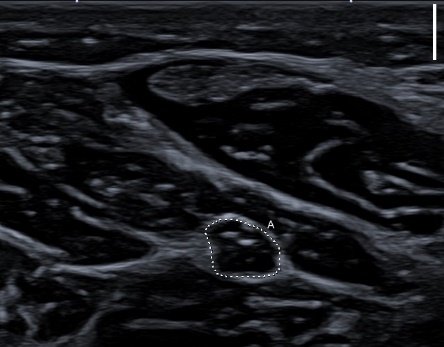 |  |
| L3 | 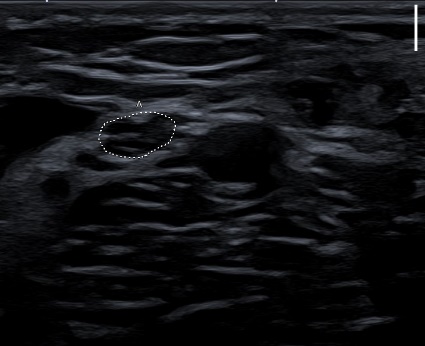 | 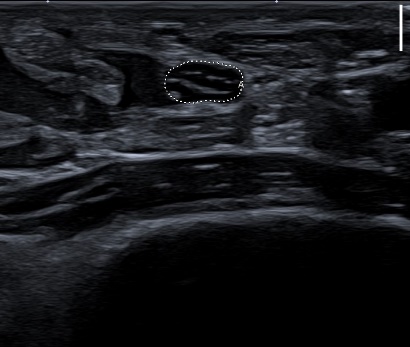 | 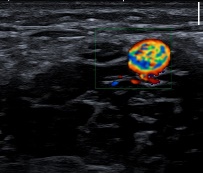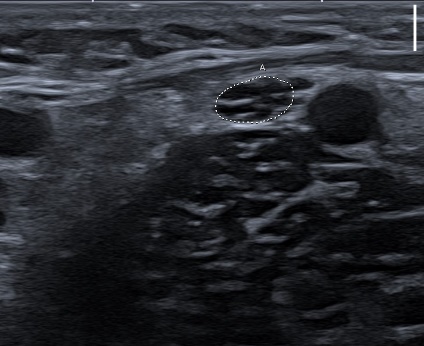 |
| R1 | 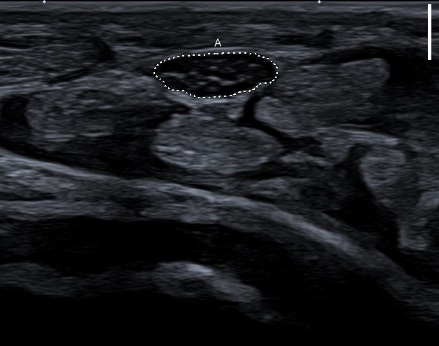 | 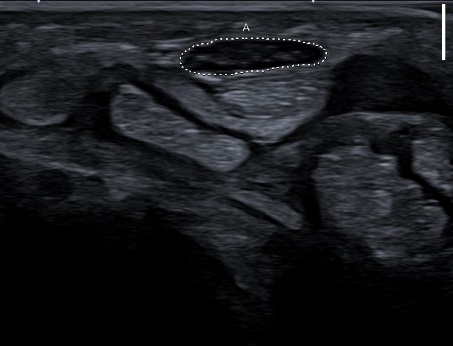 | 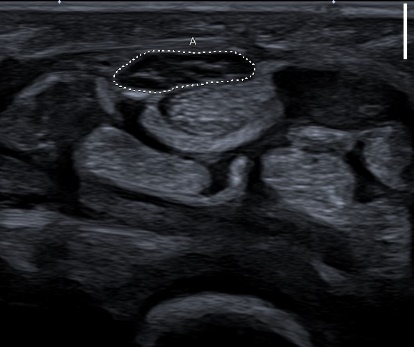 |
| R2 | 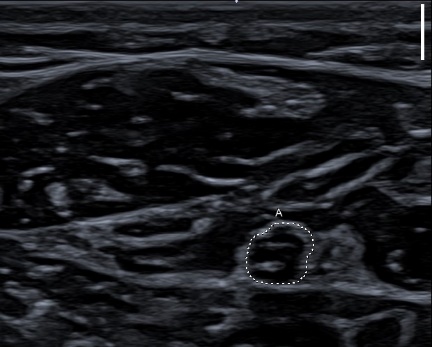 | 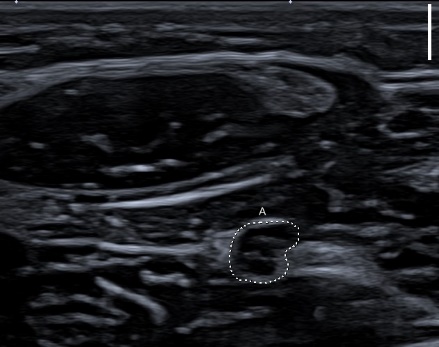 | 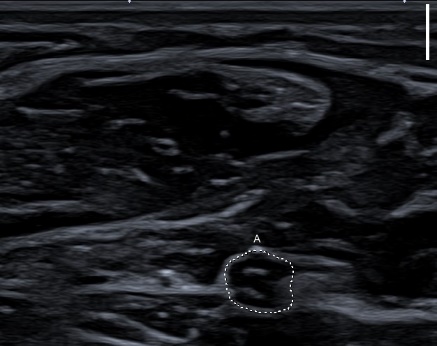 |
| R3 | 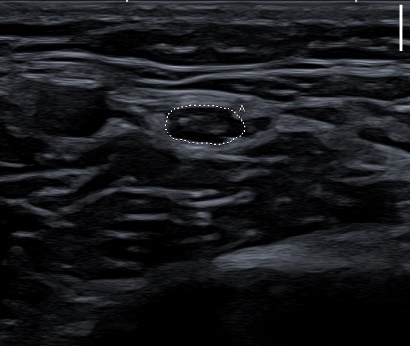 |  | 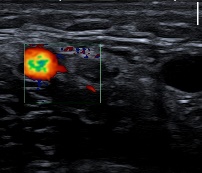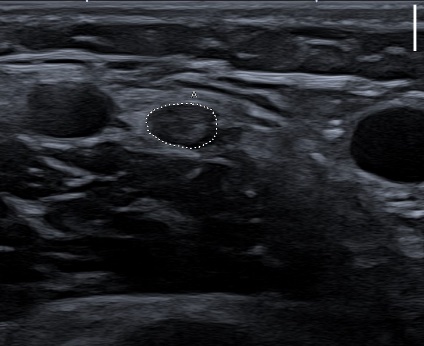 |
| Longitudinal section | | | |
| L2 |  |  | 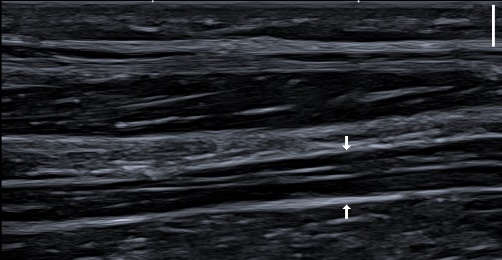 |
| R2 |  |  | 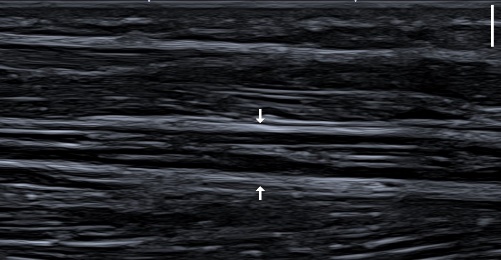 |
| 33 MHz probe | | | |
| L1 | 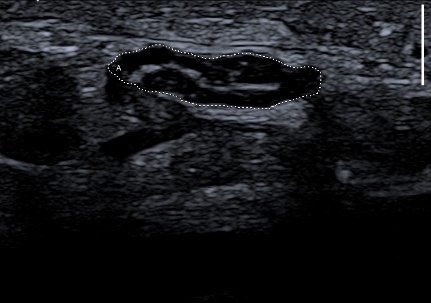 |  |  |
| R1 | 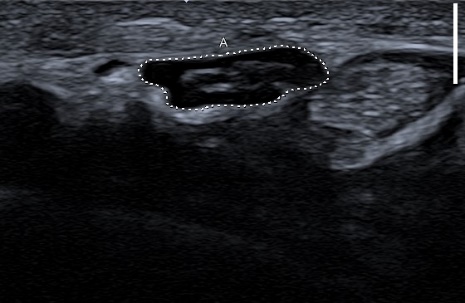 |  |  |

| **ID: 2** | | | | | | |
| --- | --- | --- | --- | --- | --- | --- |
| Site | Examination 4 | |  | |  | |
| L1 | 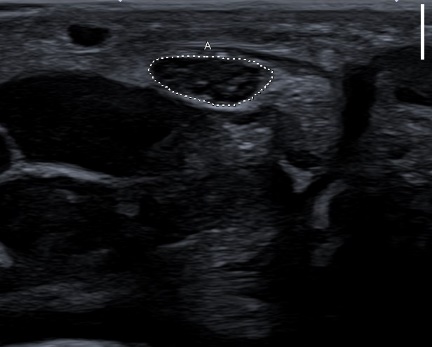 | |  | |  | |
| L2 | 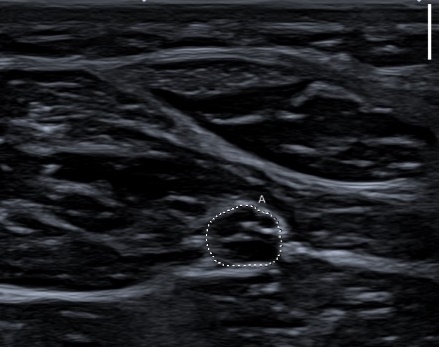 | |  | |  | |
| L3 | 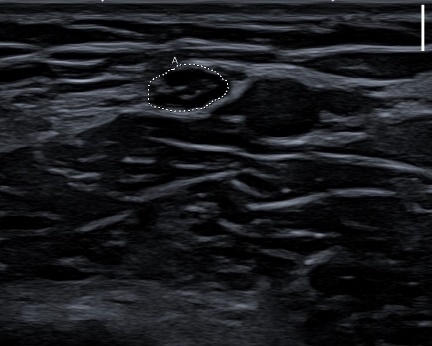 | |  | |  | |
| R1 | 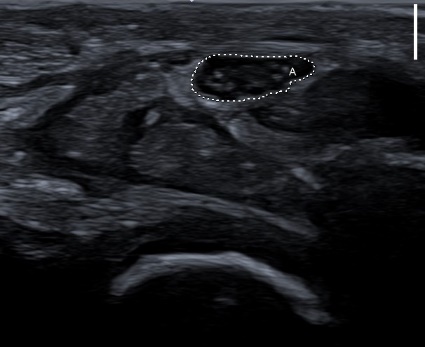 | |  | |  | |
| R2 | 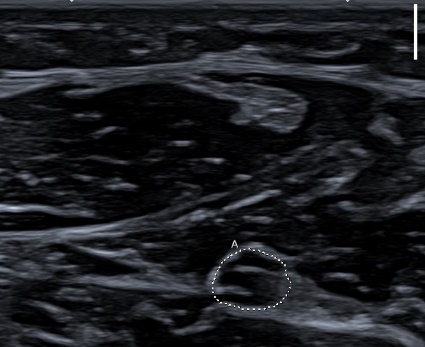 | |  | |  | |
| R3 | 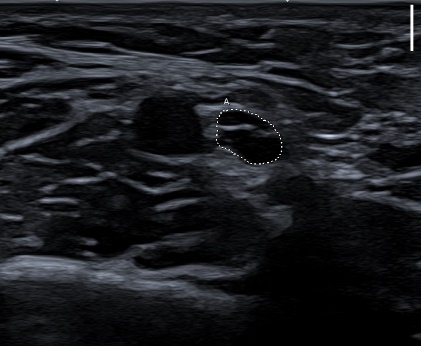 | |  | |  | |
| Longitudinal section | | | | | | |
| L2 | 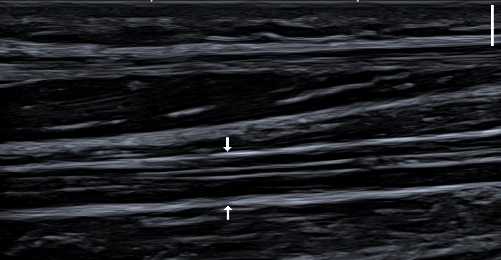 | |  | |  | |
| R2 | 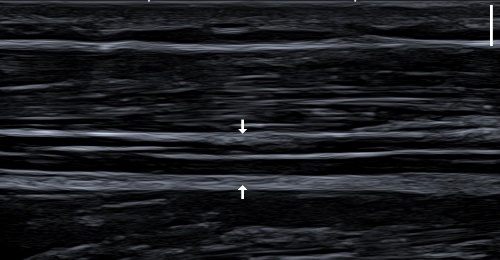 | |  | |  | |
| 33 MHz probe | | | | | | |
| L1 | | 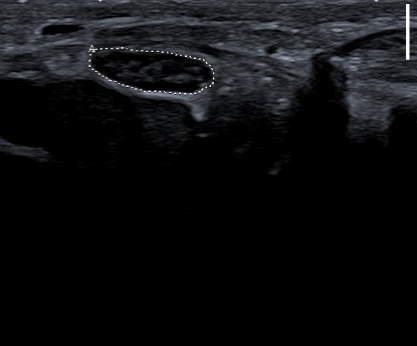 | |  | |  |
| R1 | | 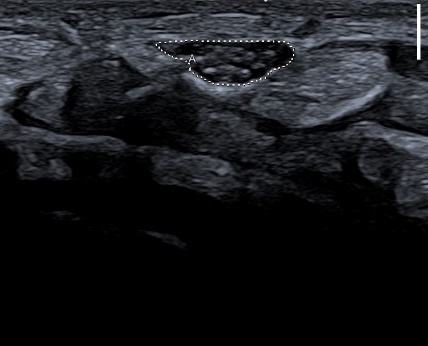 | |  | |  |

| **ID: 7** | | | | | | |
| --- | --- | --- | --- | --- | --- | --- |
| Site | Examination 1 | | Examination 2 | | Examination 3 | |
| L1 | 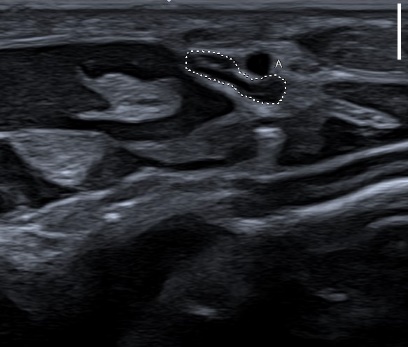 | |  | |  | |
| L2 | 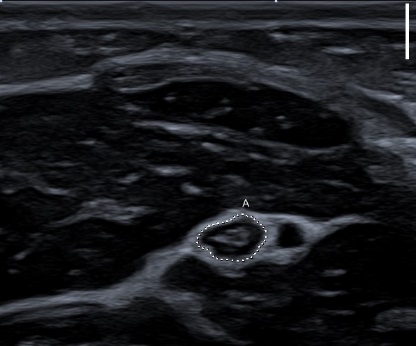 | | 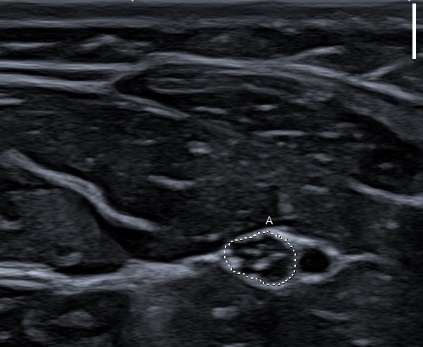 | |  | |
| L3 | 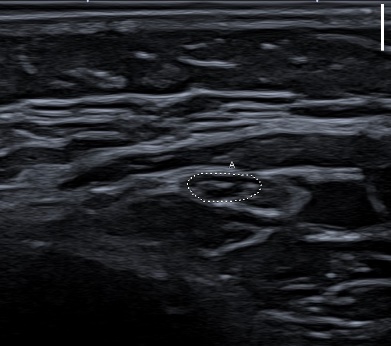 | | 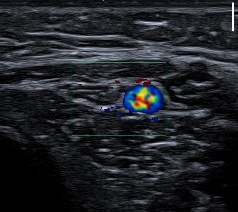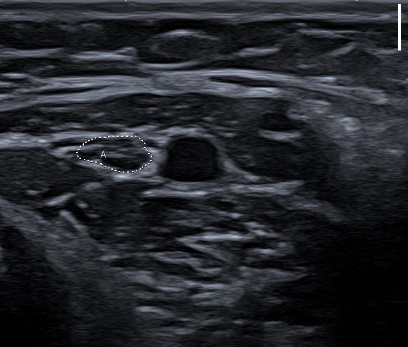 | |  | |
| R1 | 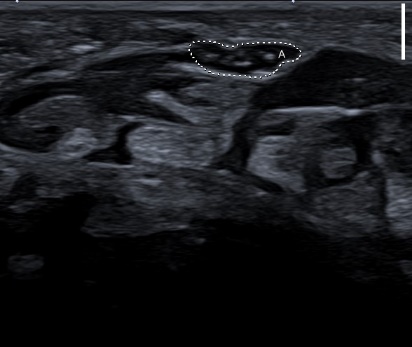 | | 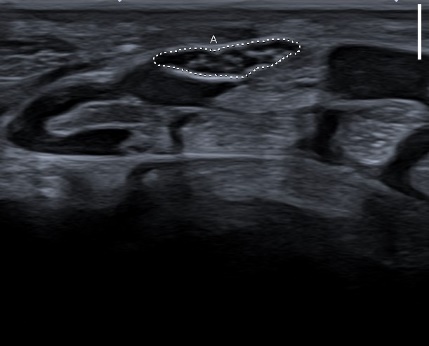 | |  | |
| R2 | 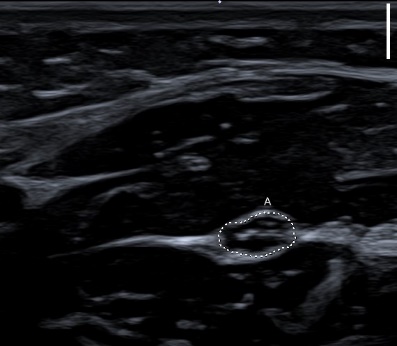 | | 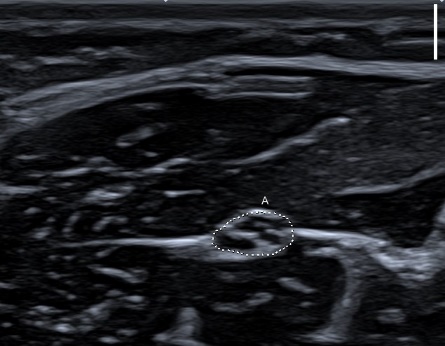 | |  | |
| R3 | 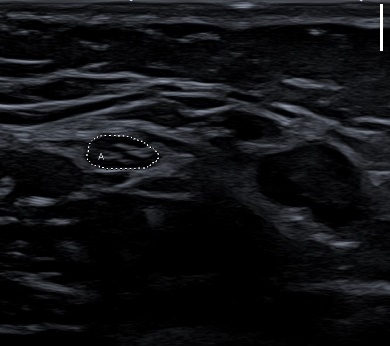 | | 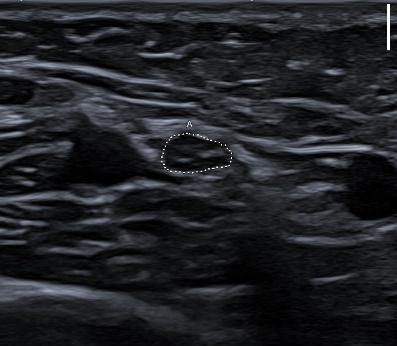 | |  | |
| Longitudinal section | | | | | | |
| L2 |  | | 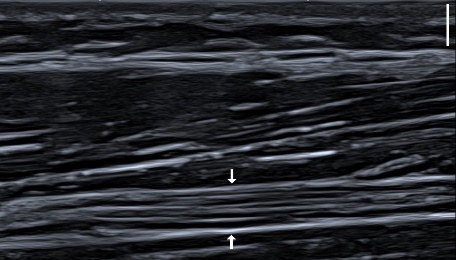 | |  | |
| R2 |  | | 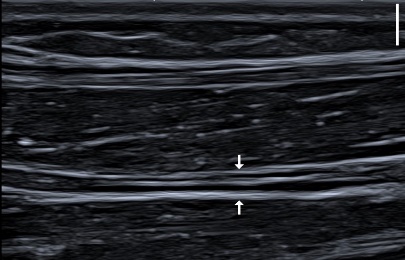 | |  | |
| 33 MHz probe | | | | | | |
| L1 | |  | |  | |  |
| R1 | |  | | 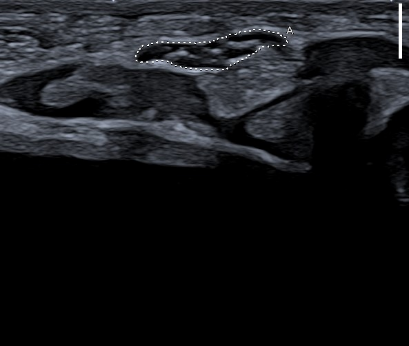 | |  |

| **ID: 8** | | | | | | | |
| --- | --- | --- | --- | --- | --- | --- | --- |
| Site | | Examination 1 | | Examination 2 | | Examination 3 | |
| L1 | | 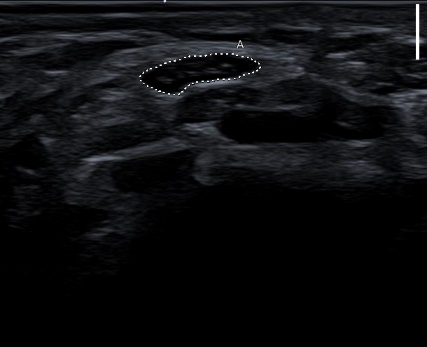 | | 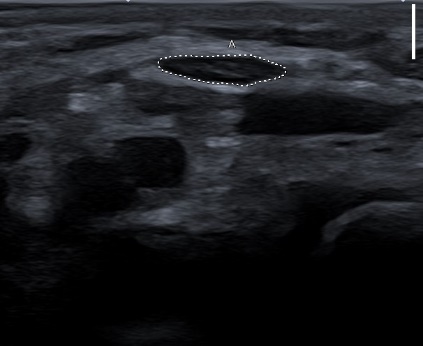 | | 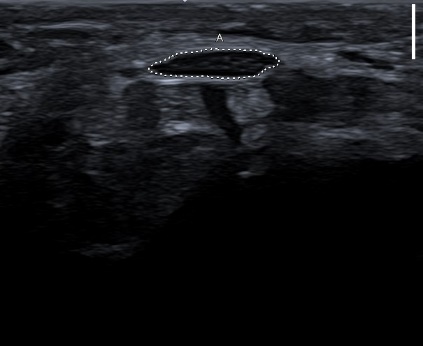 | |
| L2 | |  | | 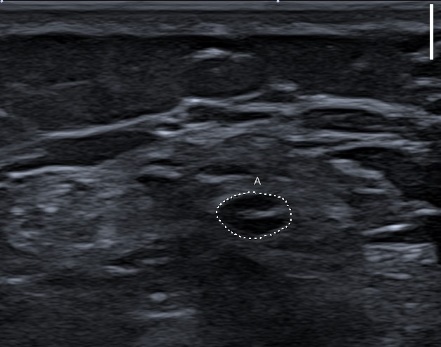 | | 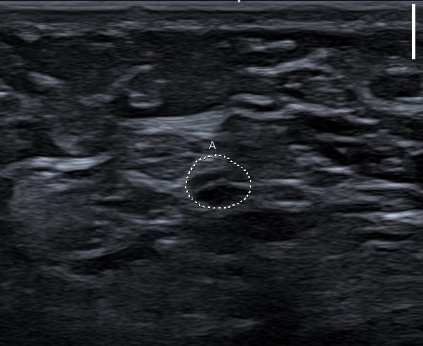 | |
| L3 | |  | | 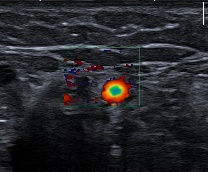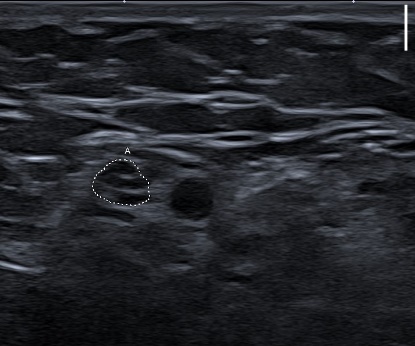 | | 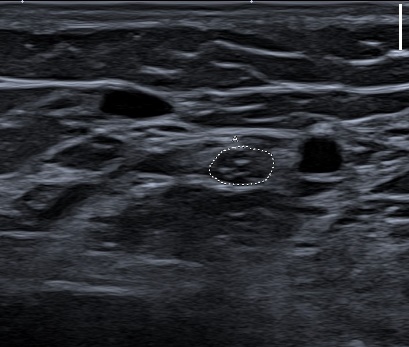 | |
| R1 | | 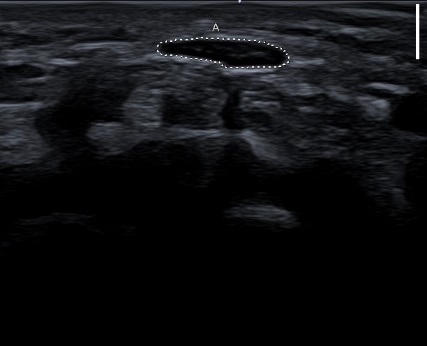 | | 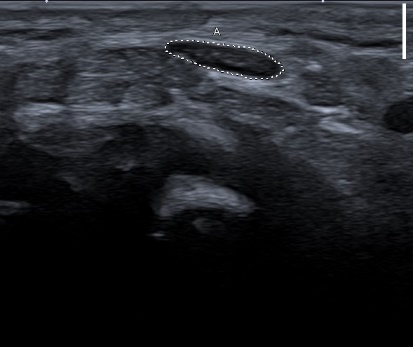 | | 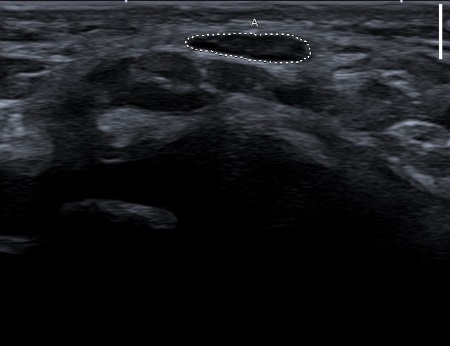 | |
| R2 | | 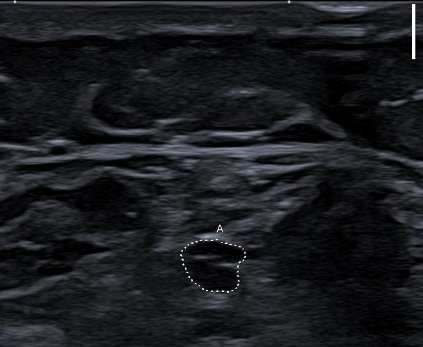 | | 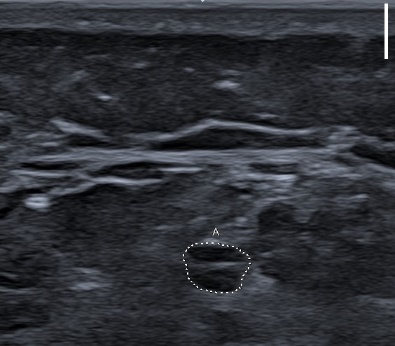 | | 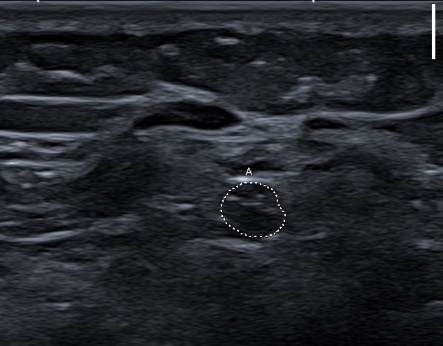 | |
| R3 | | 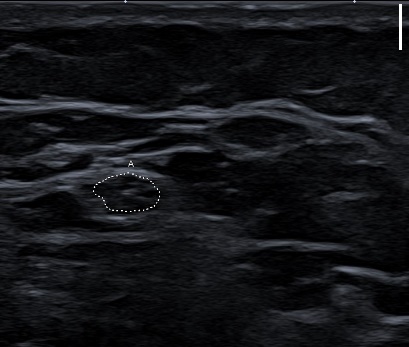 | | 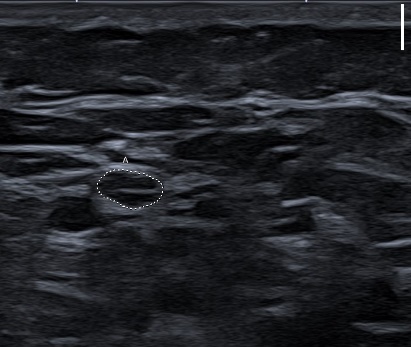 | | 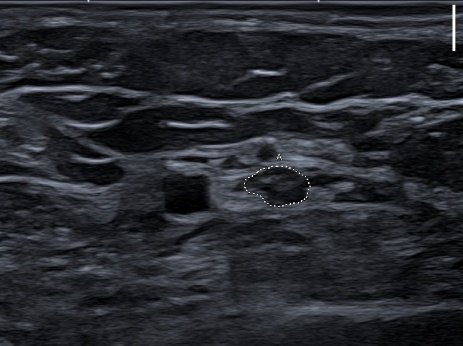 | |
| Longitudinal section | | | | | | |  |
| L2 |  | | 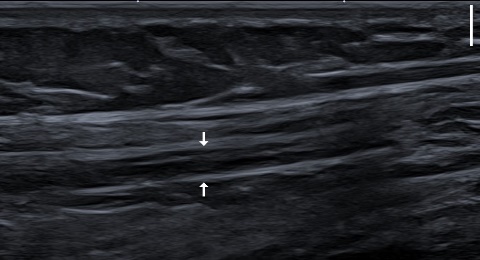 | | 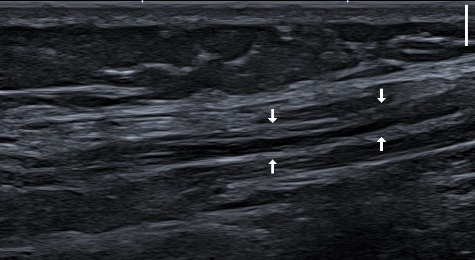 | |  |
| R2 |  | | 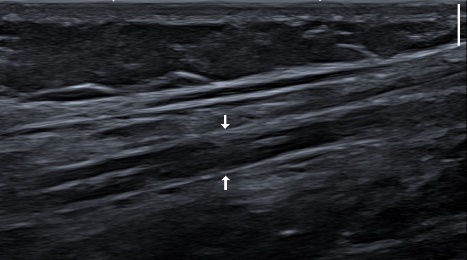 | | 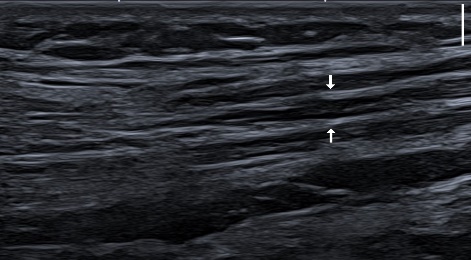 | |  |
| 33 MHz probe | | | | | | |  |
| L1 |  | | 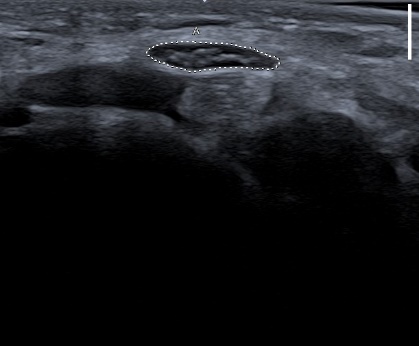 | | 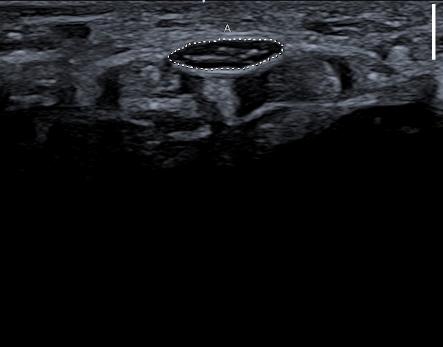 | |  |
| R1 |  | | 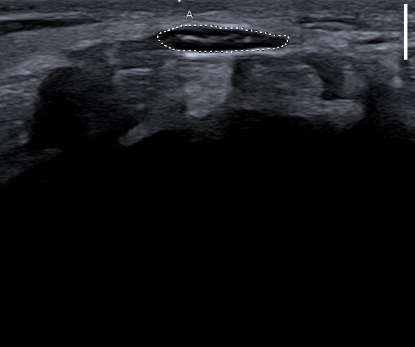 | | 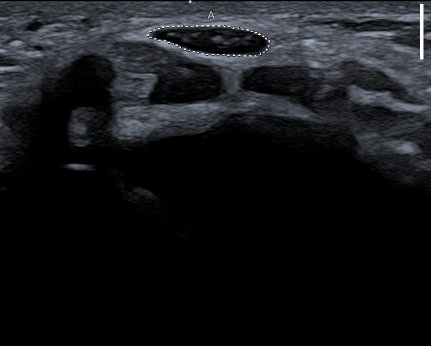 | |  |

| **ID: 6** | | | |
| --- | --- | --- | --- |
| Site | Examination 1 | Examination 2 | Examination 3 |
| L1 | 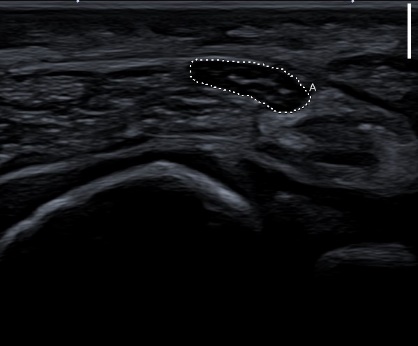 | 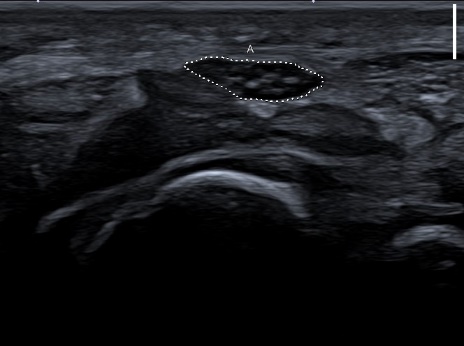 | 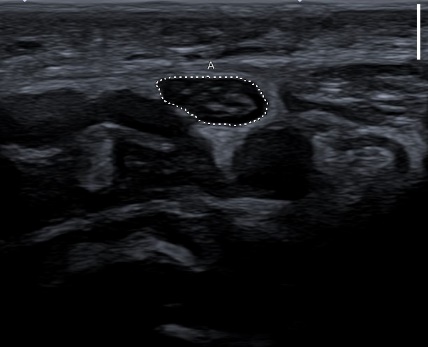 |
| L2 |  |  |  |
| L3 |  |  |  |
| R1 |  |  |  |
| R2 |  |  |  |
| R3 |  |  |  |
| Longitudinal section | | | |
| L2 |  |  |  |
| R2 |  |  |  |
| 33 MHz probe | | | |
| L1 |  |  |  |
| R1 |  |  |  |

| **ID: 12** | | | |
| --- | --- | --- | --- |
| Site | Examination 1 | Examination 2 | Examination 3 |
| L1 |  |  |  |
| L2 |  |  |  |
| L3 |  |  |  |
| R1 |  |  |  |
| R2 |  |  |  |
| R3 |  |  |  |
| Longitudinal section | | | |
| L2 |  |  |  |
| R2 |  |  |  |
| 33 MHz probe | | | |
| L1 |  |  |  |
| R1 |  |  |  |

| **ID: 3** | | | |
| --- | --- | --- | --- |
| Site | Examination 1 | Examination 2 | Examination 3 |
| L1 |  |  |  |
| L2 |  |  |  |
| L3 |  |  |  |
| R1 |  |  |  |
| R2 |  |  |  |
| R3 |  |  |  |
| Longitudinal section | | | |
| L2 |  |  |  |
| R2 |  |  |  |
| 33 MHz probe | | | |
| L1 |  |  |  |
| R1 |  |  |  |

| **ID: 11** | | | |
| --- | --- | --- | --- |
| Site | Examination 1 | Examination 2 | Examination 3 |
| L1 |  |  |  |
| L2 |  |  |  |
| L3 |  |  |  |
| R1 |  |  |  |
| R2 |  |  |  |
| R3 |  |  |  |
| Longitudinal section | | | |
| L2 |  |  |  |
| R2 |  |  |  |
| 33 MHz probe | | | |
| L1 |  |  |  |
| R1 |  |  |  |

| **ID: 1** | | | |
| --- | --- | --- | --- |
| Site | Examination 1 | Examination 2 | Examination 3 |
| L1 |  |  |  |
| L2 |  |  |  |
| L3 |  |  |  |
| R1 |  |  |  |
| R2 |  |  |  |
| R3 |  |  |  |
| Longitudinal section | | | |
| L2 |  |  |  |
| R2 |  |  |  |
| 33 MHz probe | | | |
| L1 |  |  |  |
| R1 |  |  |  |

| **ID: 4** | | | |
| --- | --- | --- | --- |
| Site | Examination 1 | Examination 2 | Examination 3 |
| L1 |  |  |  |
| L2 |  |  |  |
| L3 |  |  |  |
| R1 |  |  |  |
| R2 |  |  |  |
| R3 |  |  |  |
| Longitudinal section | | | |
| L2 |  |  |  |
| R2 |  |  |  |
| 33 MHz probe | | | |
| L1 |  |  |  |
| R1 |  |  |  |

| **ID: 10** | | | |
| --- | --- | --- | --- |
| Site | Examination 1 | Examination 2 | Examination 3 |
| L1 |  |  |  |
| L2 |  |  |  |
| L3 |  |  |  |
| R1 |  |  |  |
| R2 |  |  |  |
| R3 |  |  |  |
| Longitudinal section | | | |
| L2 |  |  |  |
| R2 |  |  |  |
| 33 MHz probe | | | |
| L1 |  |  |  |
| R1 |  |  |  |

| **ID: 9** | | | | | |
| --- | --- | --- | --- | --- | --- |
| Site | Examination 1 | Examination 2 | | | Examination 3 |
| L1 |  |  | | |  |
| L2 |  |  | | |  |
| L3 |  |  | | |  |
| R1 |  |  | | |  |
| R2 |  |  | | |  |
| R3 |  |  | | |  |
| Longitudinal section | | | | | |
| L2 |  | |  |  | |
| R2 |  | |  |  | |
| 33MHz probe | | | | | |
| L1 |  | |  |  | |
| R1 |  | |  |  | |
